# Biological Response of Human Cancer Cells to Ionizing Radiation in Combination with Gold Nanoparticles

**DOI:** 10.3390/cancers14205086

**Published:** 2022-10-17

**Authors:** Ioanna Tremi, Sophia Havaki, Sofia Georgitsopoulou, Georgia Terzoudi, Ioannis N. Lykakis, George Iliakis, Vasilios Georgakilas, Vassilis G. Gorgoulis, Alexandros G. Georgakilas

**Affiliations:** 1 DNA Damage Laboratory, Department of Physics, School of Applied Mathematical and Physical Sciences, Zografou Campus, National Technical University of Athens (NTUA), 15780 Athens, Greece; 2 Molecular Carcinogenesis Group, Department of Histology and Embryology, School of Medicine, National and Kapodistrian University of Athens, 75 Mikras Asias Street, 11527 Athens, Greece; 3 Department of Material Science, University of Patras, 26504 Patras, Greece; 4 Laboratory of Health Physics, Radiobiology & Cytogenetics, Institute of Nuclear & Radiological Sciences & Technology, Energy & Safety (INRASTES), National Centre for Scientific Research “Demokritos”, 15310 Athens, Greece; 5 Department of Chemistry, Aristotle University of Thessaloniki, University Campus, 54124 Thessaloniki, Greece; 6 Division of Experimental Radiation Biology, Department of Radiation Therapy, University Hospital Essen, University of Duisburg-Essen, 45122 Essen, Germany; 7 Institute of Medical Radiation Biology, University Hospital Essen, University of Duisburg-Essen, 45122 Essen, Germany; 8 Biomedical Research Foundation, Academy of Athens, 4 Soranou Ephessiou Street, 11527 Athens, Greece; 9 Faculty Institute for Cancer Sciences, Manchester Academic Health Sciences Centre, University of Manchester, Manchester MP13 9PL, UK; 10 Center for New Biotechnologies and Precision Medicine, Medical School, National and Kapodistrian University of Athens, 75 Mikras Asias Street, 11527 Athens, Greece; 11 Ninewells Hospital and Medical School, University of Dundee, Dundee DD1 9SY, UK

**Keywords:** gold nanoparticles (AuNPs), ionizing radiation (IR), radiosensitization, clonogenic assay, DNA damage, γH2AX, cell cycle, transmission electron microscopy (TEM), cellular senescence

## Abstract

**Simple Summary:**

Various types of metallic nanoparticles and especially gold nanoparticles (AuNPs) have been utilized in radiation studies to enhance the radiosensitization of cancer cells while minimizing detrimental effects in normal tissue. The aim of our study was to investigate the biological responses of various human cancer cells to gold-nanoparticle-induced radiosensitization. This was accomplished by using different AuNPs and several techniques in order to provide valuable insights regarding the multiple adverse biological effects, following ionizing radiation (IR) in combination with AuNPs. Insightful methodologies such as transmission electron microscopy were employed to identify comprehensively the complexity of the biological damage occurrence. Our findings confirm that AuNP radiosensitization may occur due to extensive and/or complex DNA damage, cell death, or cellular senescence. This multiparameter study aims to further elucidate the biological mechanisms and at the same time provide new information regarding the use of AuNPs as radiosensitizers in cancer treatment.

**Abstract:**

In the context of improving radiation therapy, high-atomic number (Z) metallic nanoparticles and, more importantly, gold-based nanostructures are developed as radiation enhancers/radiosensitizers. Due to the diversity of cell lines, nanoparticles, as well as radiation types or doses, the resulting biological effects may differ and remain obscure. In this multiparameter study, we aim to shed light on these effects and investigate them further by employing X-irradiation and three human cancer cell lines (PC3, A549, and U2OS cells) treated by multiple techniques. TEM experiments on PC3 cells showed that citrate-capped AuNPs were found to be located mostly in membranous structures/vesicles or autophagosomes, but also, in the case of PEG-capped AuNPs, inside the nucleus as well. The colony-forming capability of cancer cells radiosensitized by AuNPs decreased significantly and the DNA damage detected by cytogenetics, γH2AX immunostaining, and by single (γH2AX) or double (γH2AX and OGG1) immunolocalization via transmission electron microscopy (TEM) was in many cases higher and/or persistent after combination with AuNPs than upon individual exposure to ionizing radiation (IR). Moreover, different cell cycle distribution was evident in PC3 but not A549 cells after treatment with AuNPs and/or irradiation. Finally, cellular senescence was investigated by using a newly established staining procedure for lipofuscin, based on a Sudan Black-B analogue (GL13) which showed that based on the AuNPs’ concentration, an increased number of senescent cells might be observed after exposure to IR. Even though different cell lines or different types and concentrations of AuNPs may alter the levels of radiosensitization, our results imply that the complexity of damage might also be an important factor of AuNP-induced radiosensitization.

## 1. Introduction

Radiation therapy is used frequently against cancer either alone or in combination with immunotherapy or chemotherapy [1,2]. The main concern related to radiation therapy is that when applied, it does not only impact the tumor tissue, but also healthy tissue as well. Ionizing radiation (IR) leads to significant damage of cellular components directly or indirectly through the production of free radicals by water radiolysis. Radicals then react with the nearby molecules resulting in oxidation or chemical bond breakage. The most critical cellular component affected by radiation is DNA. Direct damage can lead to both DNA damage and mutations after unrepaired/misrepaired damage. IR-induced DNA damage includes single-strand breaks (SSBs) or double-strand breaks (DSBs) with the latter being the most deleterious [3]. Preventing or minimizing these damages from happening in the healthy tissue surrounding the tumor is of great importance. For this reason, attempts have been made to target or maximize the effects of radiation within the tumor area, while sparing as much as possible normal tissue areas.

In recent medical applications, nanoparticles (NPs) are considered indispensable tools in disease monitoring and therapy. Metallic nanoparticles (MNPs) of high atomic number (Z) are part of an attempt to maximize the differential response between tumor and normal tissue [4,5,6]. Gold nanoparticles (AuNPs) have demonstrated strong potential as radiosensitizers among other uses, due to their biocompatibility and the high atomic number (Z = 79) of gold, which give it a significantly high X-ray mass energy absorption coefficient compared to soft tissue [7]. The radiosensitizing effect of AuNPs is attributed mainly to a dose enhancement mechanism through the production of secondary electrons including, i.e., low energy electrons (LEEs) of 3–20 eV. These effects are maximized after exposure to low linear energy transfer (LET) radiations in the kilovoltage photon energy range [8]. More specifically, the main physical interactions when combining photon radiations with AuNPs are first the photoelectric effect which may be followed by an Auger cascade and the Compton effect. However, it is now clear that there is also a plethora of chemical and biological factors involved in the radiation enhancement action of MNPs [9,10].

Generated electrons from the surface of the AuNP react with water molecules creating free oxygen radicals, which may react with various cellular components such as mitochondria, DNA and others, and this indicates the potential of the induction of complex cellular damage [11]. Biological mechanisms of actions include oxidative stress due to reactive oxygen species (ROS) production, DNA damage or DNA repair inhibition, cell cycle effects, apoptosis or cellular senescence. The combination of IR with AuNPs can increase DNA damage quantity along with its severity and complexity. Non-resolved DNA double-strand break damage is the main cause of radiation-induced cell death. The recruitment and phosphorylation of DNA damage response protein histone H2AX acts as a marker of DNA DSB lesions. Several studies performed in various cell lines, including cancer cell lines, have indicated elevated or persistent levels of γH2AΧ or 53BP1 foci [12,13,14]. Cell cycle distribution alterations in some cases have also been evidenced after treatment with AuNPs with or without radiation. Since G2 and M phases of the cell cycle are considered the most radiosensitive, increased accumulation of cells in G2 after treatment with AuNPs is believed to increase cell radiosensitivity [15,16,17]. Most studies on cell cycle effects, however, are mostly performed in the absence of radiation and many other do not report any differences in the presence of AuNPs [18,19]. Cell death programs resulting from AuNP-induced radiosensitization are not yet clear, whereas cellular senescence has not been investigated at all. The combination of IR with AuNPs has been seen to increase apoptosis in cancer cells [20,21], but others report that AuNP radiosensitization is accompanied with autophagosome accumulation which may have other biological implications[22]. Autophagy primarily acts as a cytoprotective mechanism inside the cell; however, it has also been recognized as a cell death pathway (autophagic cell death), especially when the following criteria are met: (1) there is elevated autophagic flux, (2) there is no other programmed cell death involved, and (3) pharmacological or genetic inhibition of autophagy blocks cell death [23,24,25]. Apart from apoptosis, the presence of AuNPs inside the cell along with exposure to IR might also lead to other types of cell death [26]. Nevertheless, the outcome of autophagic cell death is still under investigation as it is important to distinguish between the cytoprotective role of autophagy and the cellular settings in which autophagy might cause cell death [25].

The physicochemical properties of gold nanoparticles, including size, shape and surface properties, are considered to affect directly the levels of radiation enhancement and are a crucial parameter of their cellular uptake and distribution inside the cell [10]. Cellular uptake and distribution are important factors of sensitivity to radiotherapy. AuNPs are located mostly inside the cytoplasm, trapped in cytoplasmic vacuoles and less frequently inside the nucleus. Even though the majority of the produced electrons are in close proximity to the NPs, dose enhancement effects are not limited to the vicinity of the NPs [27]. AuNPs accumulate preferentially in tumors in vivo [28,29] and gold is a biocompatible material and one of the optimum choices in nanomedicine. However, size, shape and surface materials of AuNPs can alter their properties, their stability, their circulation time and biodistribution. Small NPs (1–5 nm) are very good for nuclear uptake, but also sizes up to 30 nm can also pass through the nuclear pore [30]. Regarding the surface coating, various agents have been developed to improve the stability and biocompatibility of AuNPs such as polymers (e.g., polyethylene glycol (PEG)), thiols, citrate, peptides, lipids and other surfactants or inorganic coatings [31,32]. PEG is commonly used to functionalize the surface of AuNPs in order to improve their in vivo stability their biodistribution and to avoid uptake by the reticular endothelial system [33]. On the other hand, citrate is one of the most common stabilizing molecules for metal nanoparticles, giving them a negatively charged surface [34].

A large number of in vitro experiments and a much smaller number of in vivo experiments have been performed to study the AuNP-induced radiosensitization along with their dose enhancement effects [9,35,36,37,38]. Despite this, due to the dispersity of NPs, cell lines and radiation energies, the precise mechanisms are not always fully understood and therefore need to be further investigated. The use of AuNPs as radiation enhancers may increase the clustering of energy deposition events in the vicinity of the nanoparticles leading to highly complex damage in biological molecules and multiple adverse biological effects due to repair resistance of these lesions such as in the case of complex DNA damage [10]. In this study, we employed multiple techniques in order to identify a number of biological outcomes using different cancer cells after radiosensitization with gold nanoparticles mostly in the kV energy range. We used standard epithelial cancer cells lines in order to evaluate the biological responses in various human tumor tissues (i.e., prostate, lung and bone). Indirect immunofluorescence along with cytogenetics were employed for the detection of DSBs and chromatid breaks, respectively. The possibility of cell cycle distribution alterations after treatment with AuNPs with or without IR was also investigated with flow cytometry.

Transmission electron microscopy (TEM) is frequently used in order to determine AuNP uptake and localization. However, it can also be valuable in detecting DNA damage and more importantly DNA damage clustering. Until today, it has only been used by a limited number of researchers for the detection of DNA damage and specifically complex DNA damage [39,40]. Using TEM, identification and localization of different DNA repair components within the cell nuclei after radiation can be followed by the immunogold-labelling technique [41]. Regarding AuNP-induced radiosensitization and until today, TEM has only been used to determine the cellular uptake of AuNPs. In this work, TEM was used extensively in order to study the AuNP uptake and intracellular localization, structural changes and to detect and quantify DNA damage by performing both single and double immunolocalization. The phosphorylated histone protein H2AX on serine 139 (γH2AX) and the 8-oxoguanine DNA glycosylase (OGG1) were used as basic markers of DSBs and oxidative base lesions, respectively. Even though TEM has been already used for the detection and quantification of radiation-induced DNA damage and more importantly complex DNA damage [39,40], it has not been used until now in combination with AuNPs as radiosensitizers.

Finally, one of the main mechanisms identified as being involved in the biological response of cells to gold-nanoparticle-induced radiosensitization is the production of ROS and oxidative stress. Apoptosis or necrosis, as the biological outcome of IR and AuNP radiosensitization based on ROS production, has been found to be dependent on NP size and shape [42,43]. Additionally, ROS production with citrate AuNPs has been found to increase with radiation dose leading to apoptosis due to mitochondrial dysfunction [43]. In response to a variety of stresses such as radiation, mammalian cells undergo a persistent proliferative arrest known as cellular senescence. Oxidative stress and unrepaired accumulation of DNA damage can also trigger senescence. Cellular senescence in response to radiation alone has been addressed by some groups, which have reported increased senescence a few days post-irradiation with either 2, 4, or 6 Gy [44,45]. To extend this work, we tested whether treatment with AuNPs induces different levels of senescence after radiation, compared to the untreated irradiated cells. A newly established staining procedure for lipofuscin was applied. This compound is a lipophilic, biotin-linked Sudan Black B (SBB) analogue, named GL13, and has already been used extensively in both cells and tissues for the detection of senescent cells [46,47,48]. The clear purpose of this study was to investigate the biological mechanisms behind gold-nanoparticle-mediated radiosensitization by focusing on new data regarding the nuclear damage and its complexity through the use of elaborate techniques such as TEM and senescence detection.

## 2. Materials and Methods

### 2.1. Colloidal Solutions of Gold Nanoparticles

Gold nanoparticles in colloidal form were prepared using the Turkevich method [49]. A detailed description regarding the preparation of 15 nm citrate-capped AuNPs (Ct-AuNPs) and 5, 15 nm PEG-capped AuNPs (PEG-AuNPs) is described in detail in our previously published work [50]. Representative electron micrographs of the prepared AuNPs can be seen in the Supplementary Information (Section S1, Figure S1).

### 2.2. Preparation of Non-Colloidal 3% AuMTA NPs

Mesoporous TiO_2_ nanoparticle assemblies (MTA) with tunable pore size were obtained by a facile surfactant-assisted aggregating method. The pore surface of MTA is a unique host matrix (hexagonal structure) for noble metal nanoparticles which creates a new type of mesoporous Au-loaded TiO_2_ nanocomposite catalysts. The obtained materials (AuMTA) possess a continuous network of interconnected gold and TiO_2_ (~8 nm particle size) nanoparticles. Acquired 3% AuMTA NPs were loaded with 3 wt.% Au. [51,52].

### 2.3. Cell Culture

Cells were cultured in 100 mm Petri dishes at 37 °C in an atmosphere with 5% CO_2_. PC3 (human prostate cancer) cells were grown in Dulbecco’s modified MEM (DMEM) medium supplemented with 10% fetal bovine serum (FBS), 1% L-glutamine and antibiotics. A549 (human lung adenocarcinoma) and U2OS (human bone osteosarcoma) cells were grown in McCoy’s 5A medium supplemented with 10% fetal bovine serum (FBS) and antibiotics. Cells were sub-cultured every 3 days to maintain exponential growth.

### 2.4. Cell Treatment with Gold Nanoparticles

Unless specifically stated, all cells were incubated with AuNPs for 24 h at 37 °C and then washed thoroughly, at least three times with phosphate-buffered saline (PBS) to remove NPs not internalized by the cells and incubated with fresh culture medium before every radiation exposure. Cells were incubated with AuNPs at the following final concentrations: 10 μg/mL, 30 μg/mL and 100 μg/mL. Before every use, nanoparticles were resuspended in fresh culture medium in order to reach the desired final concentration. Depending on the experiment, multiple concentrations were used as dictated below in each methodology.

### 2.5. Irradiation

Cells were irradiated with an X-ray generator (GE Healthcare) operated at 320 kV, 10 mA with a 1.65 mm Al filter (effective photon energy ~90 keV), at a distance of 50 cm or 75 cm, and a dose rate of ~2.6 Gy/min or ~1.3 Gy/min, respectively. Cells were returned to the incubator at 37 °C immediately after exposure to IR, before further processing. Dosimetry was performed with a PTW and/or a chemical dosimeter, which were used to calibrate an infield ionization monitor.

### 2.6. Analysis of AuNP Cellular Uptake by Transmission Electron Microscopy (TEM)

For cellular uptake studies, PC3 cells were grown in 100 mm Petri dishes. When they reached 40–50% confluency, they were incubated with 15 nm Ct-AuNPs at a concentration of 10 μg/mL and 30 μg/mL or with 15 or 5 nm PEG-AuNPs at a concentration of 30 μg/mL for either 24 h or 48 h. After treatment with AuNPs, cells were washed thrice with PBS and fixed in 2.5% glutaraldehyde solution in 0.01 M PBS, pH 7.2–7.4 for 30 min. After fixation, cells were embedded in 4% gelatin aqueous solution and the standard procedure for TEM processing of specimens (cells–gelatin fragments) was followed, i.e., dehydration, infiltration and embedding in epoxy resin. Epoxy blocks were then cut into thin sections (~80 nm thickness), which were mounted on copper grids, stained with uranyl acetate and lead citrate, and finally observed and photographed using FEI Morgagni 268 TEM, operated at 80 kV accelerating voltage with an objective aperture of 30 μm and equipped with a digital CCD camera (Olympus Morada). The cellular uptake of 30 μg/mL Ct-AuNPs (15 nm) and PEG-AuNPs (15 nm) was quantified by using the ImageJ software. In each condition, AuNPs were counted from 30 electron micrographs at 23,000–36,000× original magnification. A very detailed description regarding TEM processing and TEM quantification analysis (ImageJ analysis) when studying the cellular uptake of gold nanoparticles can be found in our previous work [50].

### 2.7. Cell Cycle Analysis

The influence of AuNPs on the cell cycle distribution in PC3 and A549 cells was analyzed with flow cytometry with and without radiation. Exponentially growing cells were seeded in 35 mm dishes and allowed to adhere. Cells were then incubated with Ct-AuNPs (15 nm) or PEG-AuNPs (5 nm) at a 30 μg/mL final concentration in complete culture medium for 24 h. After that, cells were irradiated with 1 Gy X-rays and after that immediately placed in the incubator at 37 °C. Cells were fixed at two time points, 3 h and 24 h post IR exposure with and without AuNP treatment. Cells were fixed in 70% ice cold ethanol (ETOH, diluted in dH_2_O) after centrifugation at 1200 rpm (or 100× *g*) for 5 min and kept at 4 °C overnight. Cells were centrifuged again and resuspended in propidium iodine (PI) staining solution (40 μg/mL PI and 62 μg/mL RNase A dissolved in PBS, per $1\times 10^{6}$ cells) and left for 30 min at RT. Samples were measured in a Beckman Coulter Gallios^®^ (Brea, CA, USA) flow cytometer. For every sample, 10,000 events were analyzed by using Multicycle and standard histograms were obtained by proper gating (single cells). Obtained LMD data files were also analyzed with Kaluza^®^ (London, UK) 1.2 flow cytometry analysis software (Beckman Coulter, Brea, CA, USA).

### 2.8. Clonogenic Survival Assay

Exponentially growing PC3 and A549 cells were plated in 60 mm^2^ dishes and allowed to adhere overnight before treatment with AuNPs in complete culture medium for 24 h. Cells were incubated with 15 nm Ct-AuNPs, 5 nm PEG-AuNPs and 3% AuMTA NPs either in 30 μg/mL or 100 μg/mL final concentration, as indicated in the results below. Cells were then exposed to 0, 1, 2 and 4 Gy X-ray radiation. Cells were subsequently trypsinized, diluted and plated for colony formation. Three dishes were prepared per sample type. Cells were seeded at different plating densities for each radiation dose (ranging from 150 to 1600 cells). After 10 days of seeding, colonies were stained with 1% crystal violet in 80% methanol and counted (>50 cells/colony) under a stereomicroscope. Plating efficiency (PE) of untreated cells was calculated as ratio between colonies counted to cells seeded. Surviving fractions (SF) in irradiated cells were calculated also as ratios between colonies counted to cells seeded after correcting for PE. Data were fitted with the widely accepted linear quadratic (LQ) model:

(1) $$S=e^{-aD-\beta D^{2}}$$

and are shown as solid or dotted lines along with the data points.

### 2.9. γH2AX Indirect Immunofluorescence Assay

For immunofluorescence (IF) analysis, PC3, A549 and U2OS cells were grown on glass coverslips in 12-well plates and allowed to adhere overnight before treatment with AuNPs in complete culture medium for 24 h. For this assay, cells were incubated with 15 nm Ct-AuNPs, 5 nm PEG-AuNPs or 3% AuMTA NPs at 30 μg/mL or 100 μg/mL final concentration, as indicated also in the results below. Briefly, cells were irradiated with 1 Gy X-rays, and 30 min later, they were fixed in fixation solution (4% PFA) for 15 min at room temperature and then washed thrice over a 5 min period. Cells were permeabilized with P-solution (0.5% Triton X-100, 50 mM EDTA pH 8.0 and 100 mM Tris pH 7.4) for 10 min. After permeabilization, cells were washed twice with PBS and were blocked overnight in PBG blocking buffer (0.2% gelatin, 0.5% BSA fraction V in 1× PBS). The primary antibody (mouse monoclonal anti-γH2AX from Abcam|cat. no. ab22551) was diluted 1:800 in PBG and the samples were incubated for 1.5 h at room temperature. After three consecutive washes with PBS, samples were incubated for 1 h with the corresponding Alexa Fluor^®^ 568 Goat anti-mouse IgG secondary antibody (Life Technologies|cat. no. A4-11004, Carlsbad, CA, USA), diluted 1:400 in PBG. After two washes with PBS, samples were counterstained with 0.2 μg/mL DAPI solution, washed with PBS, and mounted in PromoFluor antifade reagent (PromoCell|cat. no. PK-PF-AFR1, Heidelberg, Germany). For each condition, 4000 cells were analyzed, and foci were detected with Zeiss Axio Scan Z1 and counted using Imaris software. The diameter of the nucleus was set at 10 μm and of the foci at 0.5 μm. A threshold was also applied to remove very small cells or cells forming clusters.

### 2.10. Transmission Electron Microscopy for the Detection of DNA Damage

PC3 cells were grown in 100 mm Petri dishes. When they reached 40–50% confluency, they were incubated with 15 nm Ct-AuNPs or with 5 nm PEG-AuNPs at a concentration of 100 μg/mL for 24 h. After 24 h incubation time, cells were washed thrice with PBS, incubated in fresh culture medium and irradiated with 1 Gy X-rays. Thirty minutes post-IR, cells were fixed in 3% paraformaldehyde and 0.5% glutaraldehyde in 0.1 M PB (sodium phosphate buffer) for 30 min. After fixation, cells were embedded in 4% gelatin aqueous solution. Specimens (cell-gelatin fragments) were then embedded in acrylic resins (Lowicryl HM20 resin from Polysciences, cat. no. 15924) by applying the progressive lowering of temperature (PLT) method [53], in order to better preserve the antigenicity for DNA damage detection. Acrylic blocks were then cut into thin sections (~80 nm thickness), which were mounted on formvar-coated Ni grids and were processed for immunogold-labelling.

Immunocytochemistry was performed using Terasaki-well plates (HLA microtest plates) with a lid to ensure a clean dust-free incubation environment and proper humidity. After blocking solution, sections were incubated with different primary antibodies, diluted 1:200 (anti-γH2AX, mouse monoclonal from Abcam, cat. no. ab22551; anti-OGG1, rabbit polyclonal from Novusbio, cat. no. NB100-106) for single or double immunolocalization, overnight at 4 °C. Immunogold labelling was applied with different gold-conjugated secondary antibodies (goat anti-mouse IgG (Gam)—10 nm from Aurion, cat. no. 810.022; goat anti-rabbit IgG (Gar)—25 nm from Aurion, cat. no. 825.011), diluted 1:40, after standardization, to give a sufficient specific signal. Afterwards, all sections were stained with uranyl acetate and lead citrate, observed and photographed using the FEI Morgagni 268 TEM microscope as previously described. Immunogold particles were counted in each condition from 60 electron micrographs at 28,000–36,000× original magnification. A detailed description on the TEM processing and TEM quantification analysis (ImageJ analysis) for DNA damage detection after incubation with gold nanoparticles can be found in our previous work [50].

### 2.11. Detection of Cellular Senescence with GL13 Staining

Exponentially growing U2OS cells were placed on glass coverslips in 60 mm dishes and allowed to adhere overnight. Then, cells were incubated with 30 μg/mL or 100 μg/mL of Ct-AuNPs (15 nm) or PEG-AuNPs (5 nm) for 24 h. After that, cells were irradiated with 4 Gy X-rays and remained in culture for 5 more days. After this period, cells were fixed with 4% (*v*/*v*) PFA solution for 10 min. To investigate senescence, a new universally applicable hybrid histo-/immunochemical method was used. In this method, cells were stained with a lipophilic, biotin-linked Sudan Black B (SBB) analogue named GL13 which offers successful and versatile senescent cell detection. GL13 staining was performed according to previous published protocol [48]. Signal development was achieved using the Novocasta Polymer Detection kit (cat. no. RE7230-K), according to the manufacturer’s instructions using DAB (brown color).

### 2.12. Statistical Analysis

Graphs were created in SigmaPlot 12.5 or Origin 8.0. Data represent the mean values ± SD based on three independent experiments. Statistical significance was determined using the Student’s *t*-test routine available in SigmaPlot 12.5. Statistical significance was indicated with asterisks as * *p* < 0.05, ** *p* < 0.01, or *** *p* < 0.001.

## 3. Results

### 3.1. Cellular Uptake of AuNPs by PC3 Cells

The use of AuNPs as radiation enhancers and their levels of radiosensitization is strongly dependent on their cellular uptake, as well as on the cellular distribution. Predominantly, in most cell lines, nanoparticles interact with the cell membrane and enter the cell mainly through energy-dependent endocytosis [54]. NPs are engulfed by the cell membrane leading to the formation of endocytic vesicles. The mechanisms responsible for their cellular uptake are macropinocytosis and receptor-mediated endocytosis such as clathrin-mediated endocytosis [55]. Even though these mechanisms are widely observed in NP uptake studies, some cell types do not possess the means necessary to perform uptake through all possible endocytotic pathways [56]. Moreover, size, shape and surface modification are parameters that directly affect the uptake level, endocytic route as well as the cytotoxicity of NPs [57,58].

We performed a qualitative along with a quantitate analysis of the cellular uptake of 15 nm Ct-AuNPs and 5 nm PEG-AuNPs by the PC3 prostate cancer cells using TEM. Cells were incubated with either type of AuNPs for 24 h, after which cells were extensively washed to remove excess or any surface-attached nanoparticles. TEM image analysis revealed numerous particles inside the cells incubated with AuNPs.

#### 3.1.1. Distribution and Localization of 15 nm Citrate-Capped AuNPs (Ct-AuNPs)

**Figure 1:**
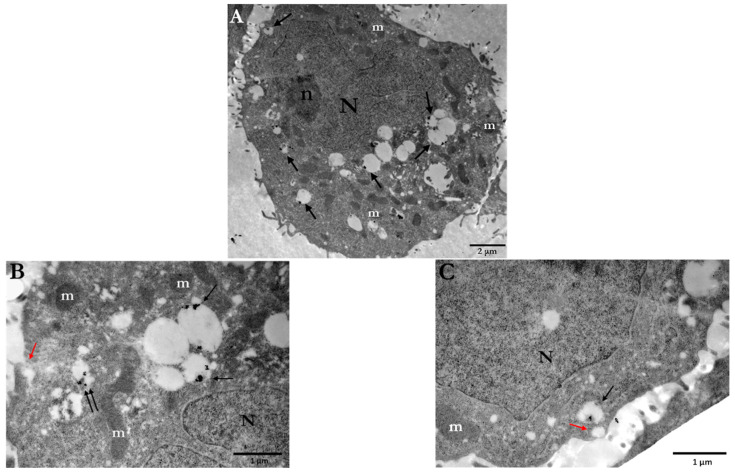
Electron micrographs of PC3 cells indicating the cellular uptake of 15 nm Ct-AuNPs (10 μg/mL) after 24 h. (**A**) Arrows point to the different areas of the cytoplasm, where nanoparticles are located inside vesicles or autophagosomes. (**B**,**C**) Higher magnification of (**A**). Ct-AuNPs are located inside vesicles (single arrows) and autophagosomes (double arrows). Red arrows indicate vesicular formations of the plasma membrane by which NPs may be endocytosed or excreted by the cell. Scale bars: (**A**) 2 μm; (**B**) and (**C**) 1 μm. N: nucleus, n: nucleolus, m: mitochondrion.

**Figure 2:**
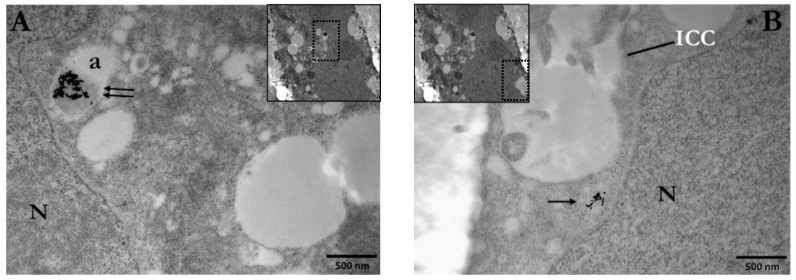
Electron micrographs of PC3 cells indicating the localization of 15 nm Ct-AuNPs (10 μg/mL) near the perinuclear region. (**A**) AuNPs are located inside an autophagosome (double arrows). (**B**) AuNPs are located inside a vesicle (single arrow). Scale bars: 500 nm. N: nucleus, a: autophagosome, ICC: intracytoplasmic canaliculus. The box with dotted line in the insets indicates the area of the cell depicted in the main figure at higher magnification.

Citrate-stabilized AuNPs are commonly used in a wide range of studies since one of the most common synthetic methods for preparation (Turkevich method) of gold nanoparticles is based on citrate reduction and stabilization, where citrate anions reduce gold ions to atoms and stabilize colloidal AuNPs [59]. PC3 cells were incubated with 10 μg/mL or 30 μg/mL of Ct-AuNPs, 15 nm in size, to study the uptake of AuNPs with increasing concentration. Inside the PC3 cells, nanoparticle agglomerates/aggregates were detected throughout the cytoplasm but the majority of AuNPs were enclosed within membranous structures/vesicles, autophagosomes, or autolysosomes, as shown in Figure 1A and at higher magnification in Figure 1B,C. In our study, vesicles were identified as single-membrane structures containing only AuNPs, whereas autophagosomes were identified as double-membrane vesicles containing NPs, as well as degraded cellular material (e.g., damaged mitochondrion, etc.). Even though Ct-AuNPs did not seem to enter the nucleus, there were often located near the perinuclear region (Figure 2A,B).

**Figure 3:**
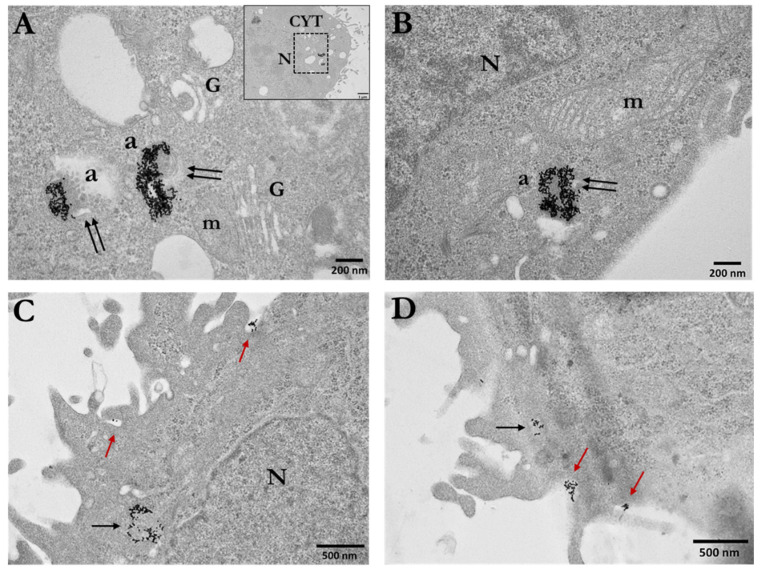
Electron micrographs of PC3 cells incubated for 24 h with 15 nm Ct-AuNPs (30 μg/mL). (**A**,**B**) Images show the distribution and localization of Ct-AuNPs in different cells. NPs were located in autophagosomes or autolysosomes (double arrows). (**C**,**D**) Nanoparticles were seen located in the cytoplasm and in vesicles (black single arrows). Red arrows indicate the mechanism by which the cell may internalize AuNPs via endocytosis. N: nucleus, m: mitochondrion, a: autophagosome/autolysosome, G: Golgi apparatus, CYT: cytoplasm. The box with dotted line in the inset of (**A**) indicates the area of the cell depicted in the main figure at higher magnification.

By increasing the concentration of Ct-AuNPs to 30 μg/mL, the number of internalized NPs increased as well. Frequently, vesicles and autophagosomes or autolysosomes containing AuNPs as agglomerates were also found near mitochondria and Golgi apparatus (Figure 3A,B). Part of our study regarding the cellular uptake of AuNPs was aiming at investigating the possible mechanisms behind this uptake. Figure 3C,D indicate areas where NPs were engulfed by the cell membrane, forming vesicular structures.

**Figure 4:**
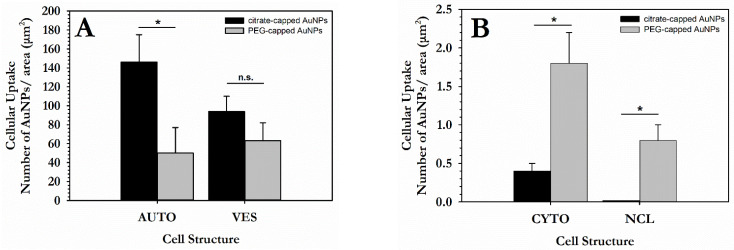
Quantification of gold nanoparticles’ (AuNPs) uptake in PC3 cells. Data are presented, in the form of histograms, as mean values of AuNPs/area in μm^2^ of different cellular compartments: (**A**) autophagosomes (AUTO), cytoplasmic vesicles (VES); (**B**) cytoplasmic area (CYTO) and nucleus (NCL). Figure shows the cellular uptake of both 15 nm Ct-capped AuNPs and PEG-capped AuNPs at 30 μg/mL concentration. Statistical significance was determined using Student *t* test: * *p* ≤ 0.05, n.s.: not significant.

For quantification purposes of the NPs’ uptake, and to provide a rudimentary overview regarding the quantity of internalized 15 nm Ct-AuNPs, we used ImageJ to analyze TEM electron micrographs. In this context, we also compared the 15 nm Ct-AuNPs with 15 nm PEG-AuNPs’ uptake, as studied previously in [50] to examine possible differences in the cellular uptake between them, due to surface modification. Results showed that even though 15 nm PEG-AuNPs were somewhat better distributed inside the cellular environment, their overall uptake after 24 h incubation was lower compared to the 15 nm Ct-AuNPs (Figure 4A,B).

#### 3.1.2. Distribution and Localization of 5 nm PEG-Capped AuNPs (PEG-AuNPs)

**Figure 5:**
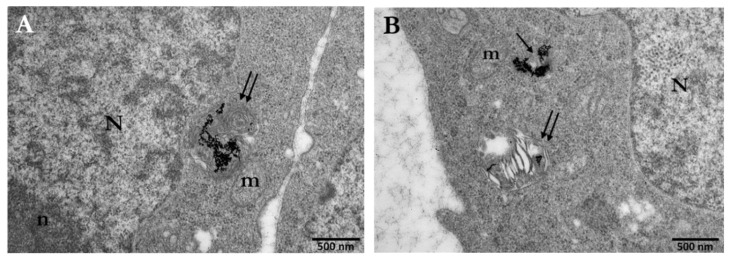

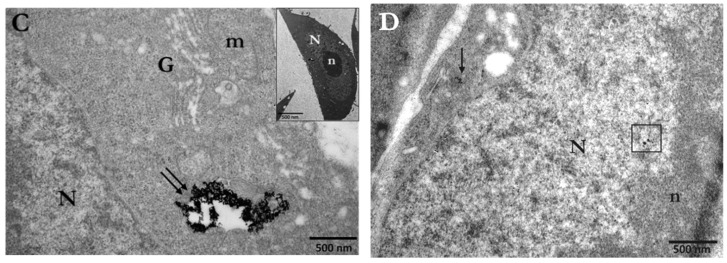
Electron micrographs of PC3 cells incubated for 24 h with 5 nm PEG-AuNPs (30 μg/mL). (**A**) Image depicts the accumulation of PEG-AuNPs inside an autophagosome (double arrows), which is located right next to the nuclear envelope. (**B**) Image depicts the localization of PEG-AuNPs in the cytoplasm (single arrows) and inside an autophagosome (double arrows) that seems to have also taken up a damaged mitochondrion. (**C**) Numerous PEG-AuNPs are located inside an autophagosome (double arrows). (**D**) Image depicts the localization of a few 5 nm PEG-AuNPs inside the nucleus (black box). N: nucleus, n: nucleolus, m: mitochondrion, G: Golgi apparatus. Scale bars = 500 nm.

A polymeric coating such as PEG provides NPs stability, which might lead to better intracellular distribution of NPs, mostly by avoiding aggregate formation. TEM experiments on the cellular uptake of 5 nm PEG-AuNPs showed a significant number of internalized nanoparticles that were located again in vesicles and autophagosomes near the perinuclear region, mitochondria and Golgi apparatus (Figure 5A–C). Moreover, PEG-AuNPs of 5 nm, were more frequently found dispersed in the cytoplasm or even inside the cell nucleus (Figure 5B,D).

**Figure 6:**
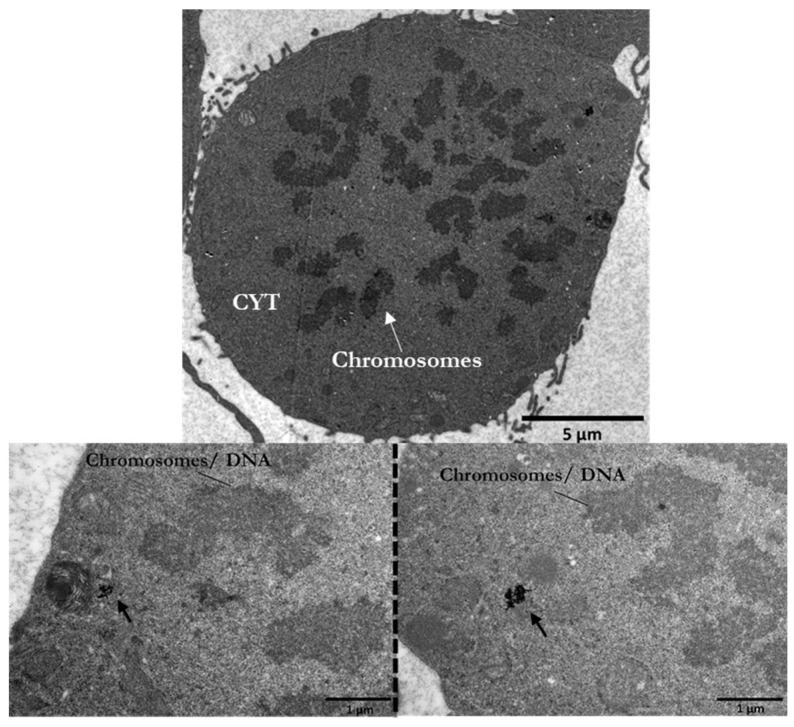
Electron micrographs of a mitotic PC3 cell after treatment with 30 μg/mL PEG-AuNPs (5 nm). After the nuclear membrane disassembles, AuNPs are much closer to the DNA. CYT: cytoplasm. Scale bars: 5 μm and 1 μm.

The localization and distance between AuNPs and the nucleus are crucial for the potential induction of DNA damage. For example, in Figure 6, a mitotic cell at the prometaphase stage is presented after treatment with 30 μg/mL of PEG-AuNPs (5 nm). The internalized AuNPs are localized very close to the chromatin structures given that the “barrier” of nuclear membrane is temporarily absent due to its disassembly, as it happens at this stage.

Cells in late G2 and mitosis (M-phase) are the most sensitive to radiation. This highlights the increased sensitivity that mitotic cells may have after radiosensitization with gold nanoparticles, due to the possible closer proximity of NPs to the DNA.

### 3.2. Different Types of AuNPs Enhanced the Response of PC3 and A549 Cells to X-ray Radiation

The AuNP-enhanced radiation responses were evaluated through a clonogenic survival assay for the different types of nanoparticles. Both A549 and PC3 cell lines demonstrated a radiosensitization effect compared to those exposed only to IR. A549 cells were treated with Ct-AuNPs (15 nm), PEG-AuNPs (5 nm) and 3% AuMTA NPs at a 30 μg/mL or 100 μg/mL final concentration.

**Figure 7:**
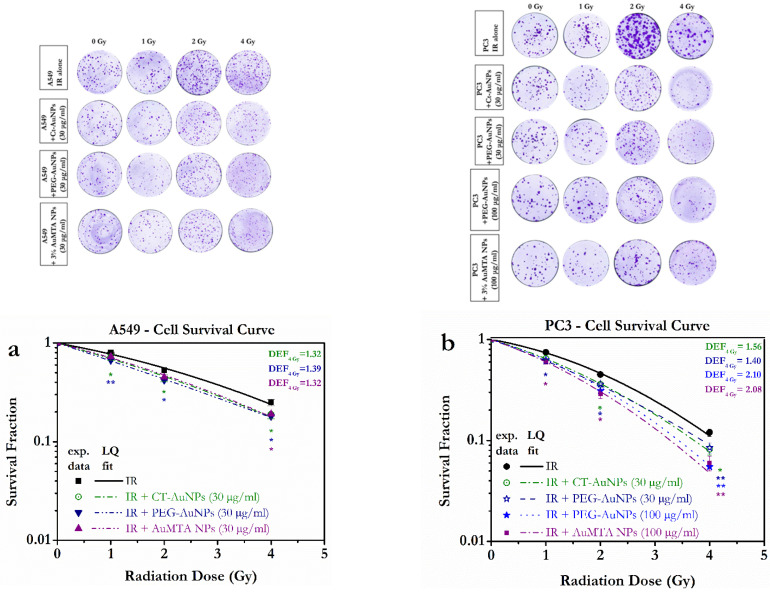
Radiosensitization of A549 (**a**) and PC3 cells (**b**) by gold nanoparticles. (**a**,**b**) represent the normalized survival curves for A549 and PC3 cells irradiated with 320 KVp X-rays (denoted ‘IR’ for the untreated groups). Data were fitted based on the linear quadratic (LQ) model. The DEFs for the dose of 4 Gy are also presented in the graphs. Statistical significance was determined using Student *t* test: * *p* ≤ 0.05, ** *p* ≤ 0.01. Upper images show indicatively the colony formation for the untreated A549 and PC3 cells and for the A549 and PC3 cells treated with AuNPs, at radiation doses 1.2 and 4 Gy.

A549 cells did not exhibit very high levels of radiosensitization by AuNPs, and were also more radioresistant overall as can be seen in Figure 7a. The radiosensitivity enhancement ratio, or else DEF with AuNPs, was higher for 2 (not shown here) and 4 Gy and in general, it increased with increasing the dose, for all types of AuNPs. PEG-AuNPs (5 nm) showed the higher levels of radiosensitization especially at 4 Gy, with a DEF_4Gy_ = 1.39, and led to a reduced colony formation, compared to the irradiated-alone A549 cells.

Correspondingly, PC3 cells were treated with Ct-AuNPs (15 nm), PEG-AuNPs (5 nm) and 3% AuMTA NPs, both at low (30 μg/mL) and high concentrations (100 μg/mL). The results were similar to the A549 cells when the lower concentration of AuNPs was used, but PC3 cells were in general more radiosensitive and showed enhanced radiosensitivity when treated with AuNPs. Radiosensitization levels can be quite dependent on the NP concentration and so by increasing the concentration of PEG-AuNPs (5 nm) and 3% AuMTA NPs, we noticed that the overall radiosensitivity increased as well. PC3 cells treated with 100 μg/mL of PEG-AuNPs or 3% AuMTA NPs exhibited significantly increased enhancement ratios at 4 Gy (DEF_4Gy_ = 2.10 and DEF_4Gy_ = 2.08, respectively). Although, radiosensitization with AuNPs was abundant in all doses as can be seen in Figure 7b.

While fitting the data to the LQ equation, the α and β constants were also calculated and are presented in Tables S1 and S2 (Supplementary Information, Section S2).

### 3.3. Assessment of the Enhancement of DNA DSBs in Cells with Internalized Gold Nanoparticles

γH2AX protein, which is present at the sites of DSBs, was probed for the detection and quantification of DNA damage. 

**Figure 8:**
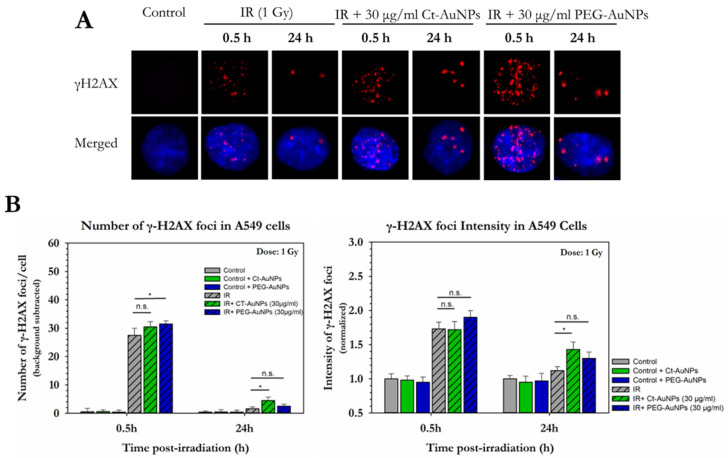
Quantitative and qualitative representation of DSBs in A549, after AuNP-induced radiosensitization with 1 Gy 320 KVp X-rays. (**A**) The nucleus is stained with DAPI, shown in blue, and the marker for DNA DSBs (γH2AX) is shown in red. (**B**) Figures show the average number of γH2AX foci as well as the normalized foci intensity counted 0.5 h and 24 h after exposure. Statistical significance was determined using Student *t* test: * *p* ≤ 0.05, n.s.: not significant.

A549 cells did not show significant changes in the levels of DSBs 0.5 h or 24 h after 1 Gy of IR exposure (Figure 8A,B). However, cells treated with 30 μg/mL of PEG-AuNPs showed an increase in the formation of γH2AX foci compared to the irradiated-only cells, 0.5 h after exposure (31.5 γH2AX foci vs. 27, respectively), while going back to normal after 24 h. Additionally, cells treated with 30 μg/mL of Ct-AuNPs had increased remaining foci after 24 h, compared to the irradiated only cells (1.6 γH2AX foci vs. 4.5, respectively). In the non-irradiated A549 cells (controls), there was no change in the γH2AΧ foci formation when cells were treated with or without AuNPs and the mean number of foci ranged between 2.5 and 4. The mean number of γH2AΧ foci in the control groups was subtracted from the corresponding irradiated conditions. Regarding the intensity of the γH2AX foci, no significant differences were observed 0.5 h after IR exposure, when cells were treated with AuNPs. Again, however, there was an increase in the foci intensity of the cells treated with 30 μg/mL of Ct-AuNPs 24 h after exposure compared to the untreated irradiated cells (1.43 vs. 1.12, respectively).

**Figure 9:**
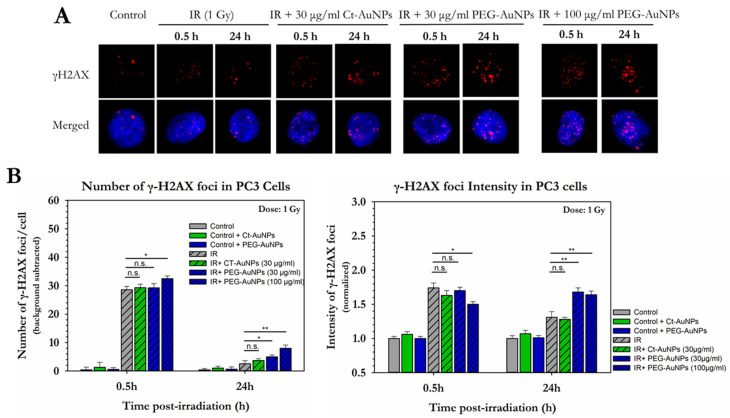
Quantitative and qualitative representation of DSBs in PC3 cells, after AuNP-induced radiosensitization with 1 Gy 320 KVp X-rays. (**A**) The nucleus is stained with DAPI, shown in blue, and the marker for DNA DSBs (γH2AX) is shown in red. (**B**) Figures show the average number of γH2AX foci as well as the normalized foci intensity counted 0.5 h and 24 h after exposure. Statistical significance was determined using Student *t* test: * *p* ≤ 0.05, ** *p* ≤ 0.01, n.s.: not significant.

Surprisingly, PC3 cells 0.5 h after 1 Gy of IR exposure did not show significant increase in the formation of γH2AX foci compared to the irradiated-only cells (Figure 9A,B). Since PC3 cells were found to be more radiosensitive in the clonogenic assay and since 5 nm PEG-AuNPs are able to enter the nucleus, we also investigated the level of DNA damage after treatment with 100 μg/mL PEG-AuNPs. In this case, there was increased formation of DSBs 0.5 h after 1 Gy of IR exposure, compared to the irradiated-only cells (32.5 γH2AX foci vs. 28.5, respectively). More importantly, cells treated with both concentrations of PEG-AuNPs exhibited higher numbers of residual γH2AX foci 24 h after exposure suggesting repair resistance. Residual foci 24 h post-IR also exhibited higher intensity values. The mean intensity for the cells treated with 30 μg/mL was 1.68 and for the cells treated with 100 μg/mL was 1.64, whereas for the untreated irradiated cells, it was 1.3. What is also worth noting is that the intensity of the foci for the groups treated with PEG-AuNPs remained almost the same 24 h after exposure compared to 0.5 h.

**Figure 10:**
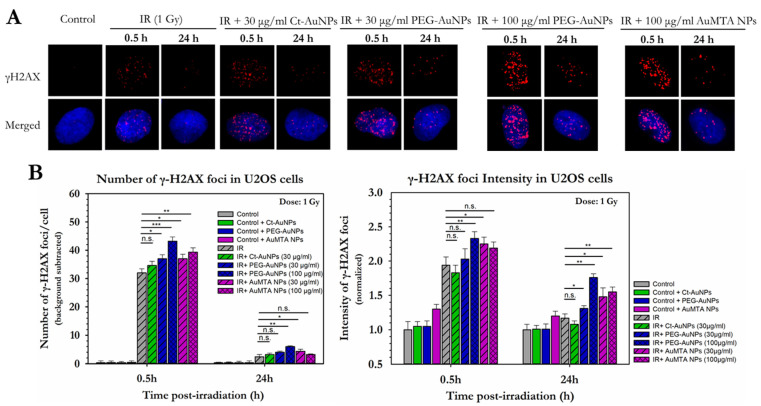
Quantitative and qualitative representation of DSBs in U2OS cells, after AuNP-induced radiosensitization with 1 Gy 320 KVp X-rays. (**A**) The nucleus is stained with DAPI, shown in blue, and the marker for DNA DSBs (γH2AX) is shown in red. (**B**) Figures show the average number of γH2AX foci as well as the normalized foci intensity counted 0.5 h and 24 h after exposure. Statistical significance was determined using Student *t* test: * *p* ≤ 0.05, ** *p* ≤ 0.01, *** *p* ≤ 0.001, n.s.: not significant.

To extend this further and due to the dispersity of the cancer cell lines in response to induced DNA damage, we also investigated DSBs in U2OS cells after treatment with AuNPs (Figure 10A,B) U2OS cells showed increased numbers of γH2AX foci 0.5 h after 1 Gy of IR exposure in all the groups treated with PEG-AuNPs, except Ct-AuNPs. More specifically, irradiated cells with 1 Gy gave 32 γH2AX foci 0.5 h after exposure. At the same time, cells treated with 30 μg/mL PEG-AuNPs gave 37 γH2AX foci, while cells treated with 100 μg/mL PEG-AuNPs gave 43 γH2AX foci. Due to the obvious sensitivity of this cell line, we also investigated DSBs after treatment with 30 μg/mL and 100 μg/mL of 3% AuMTA NPs, which gave 37 and 39.5 γH2AX foci, respectively. Regarding the intensity, cells treated with 100 μg/mL PEG-AuNPs and 30 μg/mL 3% AuMTA NPs, 0.5 h after exposure gave foci of increased intensity 2.33 and 2.25, respectively, compared to the untreated irradiated cells (1.93). After 24 h all the groups treated with AuNPs exhibited higher intensity foci compared to the untreated irradiated cells which was found to be 1.17. Specifically, cells treated with 30 μg/mL PEG-AuNPs and 100 μg/mL PEG-AuNPs gave foci with intensities 1.31 and 1.76, respectively, while cells treated with 30 μg/mL 3% AuMTA NPs and 100 μg/mL 3% AuMTA NPs gave intensity values of 1.48 and 1.55, respectively.

### 3.4. Cell Cycle Distribution of A549 and PC3 Cells after AuNP-Induced Radiosensitization

The sensitivity and biological effects of radiation exposure are dependent upon the cell cycle phase. In order to investigate possible cell cycle phase alterations, PC3 and A549 cells were treated with Ct-AuNPs (15 nm) and PEG-AuNPs (5 nm) at 30μg/mL final concentration and then irradiated with 1 Gy. Cell cycle distribution was evaluated 3 h and 24 h after radiation exposure.

**Figure 11:**
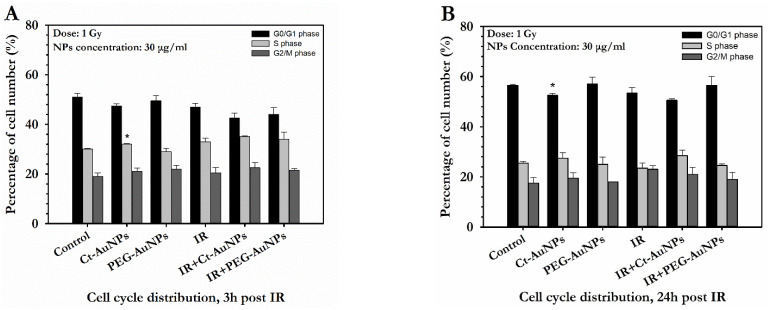
Flow cytometry analysis of cell cycle phase distribution of A549 cells in dependence of the irradiation and AuNP treatment (30 μg/mL). (**A**) Cell cycle distribution 3 h after 1 Gy of IR exposure. Cells were treated with AuNPs for 24 h, irradiated and collected 3 h after exposure. (**B**) Cell cycle distribution 24 h after 1 Gy of IR exposure. Cells were treated with AuNPs for 24 h, irradiated and collected 24 h after exposure. Statistical significance was determined using Student *t* test: * *p* ≤ 0.05. Non-irradiated groups with AuNPs were compared with the control group to evaluate statistical significance whereas irradiated groups with AuNPs were compared with the radiation alone group.

A549 cells did not exhibit any significant differences with and without treatment with AuNPs. Additionally, in the majority, no differences were observed between the irradiated cells with and without AuNPs at either 3 h or 24 h after IR exposure (Figure 11A,B).

**Figure 12:**
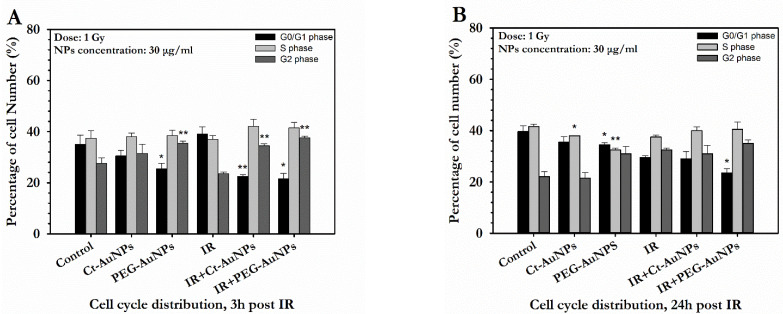
Flow cytometry analysis of cell cycle phase distribution of PC3 cells in dependence of the irradiation and AuNP treatment (30 μg/mL). (**A**) Cell cycle distribution 3 h after 1 Gy of IR exposure. Cells were treated with AuNPs for 24 h, irradiated and collected 3 h after exposure. (**B**) Cell cycle distribution 24 h after 1 Gy of IR exposure. Cells were treated with AuNPs for 24 h, irradiated and collected 24 h after exposure. Statistical significance was determined using Student *t* test: * *p* ≤ 0.05, ** *p* ≤ 0.01. Non-irradiated groups with AuNPs were compared with the control group to evaluate statistical significance whereas irradiated groups with AuNPs were compared with the radiation alone group.

In contrast to the A549 cells, non-irradiated PC3 cells showed decreased number of cells in G1 phase and increased number of cells in G2 phase after treatment with PEG-AuNPs. The same result was also observed in combination with radiation (Figure 12A,B). On the other hand, while treatment with Ct-AuNPs did not cause any significant differences in the cell cycle distribution in the absence of radiation (*p* > 0.05), PC3 cells treated with Ct-AuNPs showed an increased number of cells in G2 phase when combined with radiation, at 3 h after IR exposure.

Moreover, irradiated PC3 cells treated with PEG-AuNPs still exhibited a decreased number of cells in the G1 phase 24 h after IR exposure. The corresponding flow cytometry histograms for both A549 and PC3 cells can be found in the Supplementary Information (Section S3, Figures S2 and S3).

### 3.5. DNA Damage Detection and Quantification in PC3 Cells by TEM

Antibodies conjugated with gold particles provide specific and precise targeting of biological markers and are used to localize antigens in tissues and cells for subsequent imaging using a transmission electron microscope. We have previously introduced the use of the immunogold-labelling technique to study gold nanoparticle radiosensitization applying both single and double immunolocalization [50]. Here, we investigated in PC3 cells the formation of DSBs by applying single immunolocalization of γH2AX, as well as the formation of DSBs and base lesions (such as the 8-oxoguanine) due to oxidative stress from ROS production, by applying double immunolocalization of γH2AX and OGG1, respectively. For γH2AX detection, we used a secondary antibody conjugated to 10 nm immunogold particles, whereas for OGG1 detection, we used a secondary antibody conjugated to 25 nm immunogold particles.

**Figure 13:**
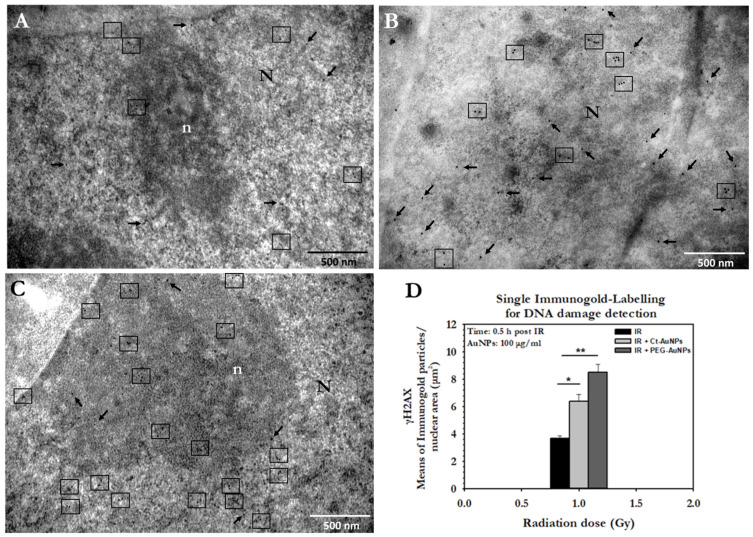
Representative electron micrographs and quantification of single immunolocalization in PC3 cells for γH2AX detection inside the nucleus, 0.5 h after 1 Gy of IR exposure. (**A**) Image depicts the detection of γH2AX after irradiation, but without treatment with AuNPs. (**B**) Image depicts the detection of γH2AX after gold-nanoparticle-induced radiosensitization with 100 μg/mL Ct-AuNPs (15 nm). (**C**) Image depicts the detection of γH2AX after gold-nanoparticle-induced radiosensitization with 100 μg/mL PEG-AuNPs (5 nm). N: nucleus, n: nucleolus. Arrows in each image indicate γH2AX single immunogold particles and boxes are γH2AX clusters, the formation of which was increased after treatment with gold nanoparticles, compared to the irradiated-only cells. Scale bars = 500 nm. (**D**) Number of DSBs induced by 1 Gy X-rays in PC3 cells, both with and without treatment with AuNPs. γH2AX marker was used for the detection of DSBs and analysis was performed by TEM analysis and Image J. Results indicate mean number of γH2AX particles per nuclear area μm^2^. Statistical significance was determined using Student *t* test: * *p* ≤ 0.05, ** *p* ≤ 0.01.

Single γH2AX immunogold localization was performed in PC3 cells incubated with either 15 nm Ct-AuNPs or 5 nm PEG-AuNPs, at a 100 μg/mL concentration. Representative electron micrographs of the single immunolocalization for all conditions are presented below (Figure 13A–C).

The number of DSBs was also quantified by image analysis. The results are presented as the number of immunogold particles (gold-conjugated antibodies detecting γH2AX) per nuclear area in μm^2^. PC3 cells treated with 100 μg/mL Ct-AuNPs (15 nm) showed an increased number of γH2AX particles 0.5 h after 1 Gy irradiation compared to the irradiated cells that were not previously treated with AuNPs. However, cells treated with the same concentration of PEG-AuNPs (5 nm) exhibited an even higher number of γH2AΧ particles (Figure 13D). This increase might be due to the fact that 5 nm PEG-AuNPs can also be found at a smaller extent inside the nucleus, apart from the cytoplasmic areas. Small AuNPs ≤ 5 nm in diameter are considered the most potent radiosensitizers in terms of NP size as they present a greater surface-area-to-volume ratio.

**Figure 14:**
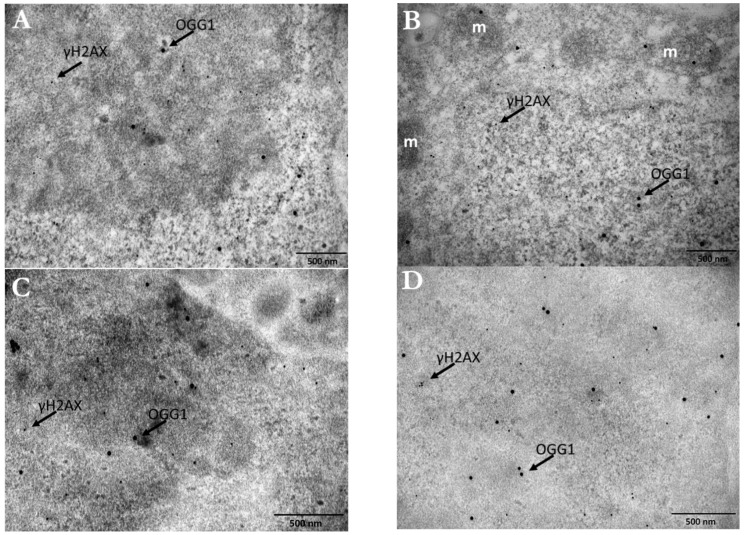
Electron micrographs of PC3 cells after immunogold labelling for DSBs and oxidative lesion detection. Images represent double immunolocalization for γH2AX and OGG1 detection inside the nucleus, 0.5 h after 1Gy of IR exposure. The size of immunogold particles used for γH2AX and OGG1 was 10 nm and 25 nm, respectively. Arrows in each image indicate the γH2AX and OGG1 particles. (**A**,**B**) Images depict the detection of γH2AX and OGG1 after irradiation but without treatment with AuNPs. m: mitochondrion. (**C**,**D**) Images depict the detection of γH2AX and OGG1 after induced radiosensitization with 100 μg/mL Ct-AuNPs (15 nm).

Double immunolocalization was performed only in PC3 cells treated with 15 nm Ct-AuNPs, due to the fact that these NPs were located only in the cytoplasm and, therefore, the distinction between the AuNPs used for radiosensitization and the different sizes of gold-conjugated antibodies was easier and well-defined. Representative electron micrographs of the double immunolocalization are presented below (Figure 14A–D).

**Figure 15:**
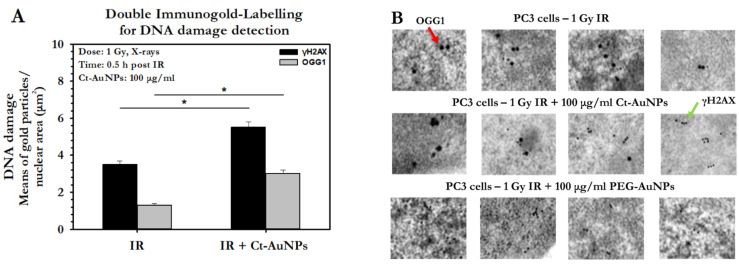
Formation of DSBs and oxidative lesions after treatment with AuNPs and X-ray irradiation. (**A**) Number of DSBs and 8-oxoguanine base lesions, induced after 1 Gy of IR exposure in PC3 cells, both with and without treatment with 100 μg/mL Ct-AuNPs (15 nm). γH2AX marker was used for the detection of DSBs and OGG1 was used for the detection of oxidative base lesions. Results indicate mean number of γH2AX particles per nuclear area μm^2^. Statistical significance was determined using Student *t* test: * *p* ≤ 0.05. (**B**) Electron micrographs indicating the increased formation of DNA damage clusters in PC3 cells, after gold-nanoparticle-induced radiosensitization. Red and green arrows point to the different sizes of immunogold particles depicting OGG1 (25 nm) and γH2AX (10 nm) markers, respectively.

The number of 10 nm and 25 nm immunogold particles detecting the γH2AX and OGG1 antigenic sites, respectively, was quantified by image analysis. Results showed increased number of γH2AX particles/nuclear area (μm^2^) after the AuNPs’ radiosensitization, but more importantly, the number of OGG1 particles/nuclear area (μm^2^) was doubled after induced radiosensitization with 100 μg/mL Ct-AuNPs, even at 30 min post-IR exposure (Figure 15A). In the context of the TEM study, we also noticed that the formation of DNA damage clusters was more prominent in cells treated with AuNPs and then irradiated, compared to the irradiated-only cells. Some representative images can be seen below in Figure 15B.

### 3.6. Detection of Cellular Senescence after AuNP-Induced Radiosensitization

Gold nanoparticles, in combination with ionizing radiation, contribute to increased radiosensitization through which enhanced radical production has been observed after irradiation and enhanced oxidative stress. It is known that the action of IR can even lead to cellular senescence. Regarding the different cellular responses and cellular outcomes, the possible induction of cellular senescence due to gold-nanoparticle-induced radiosensitization has not been investigated to date. This induction could be caused by a variety of factors such as prolonged oxidative stress and DNA damage.

**Figure 16:**
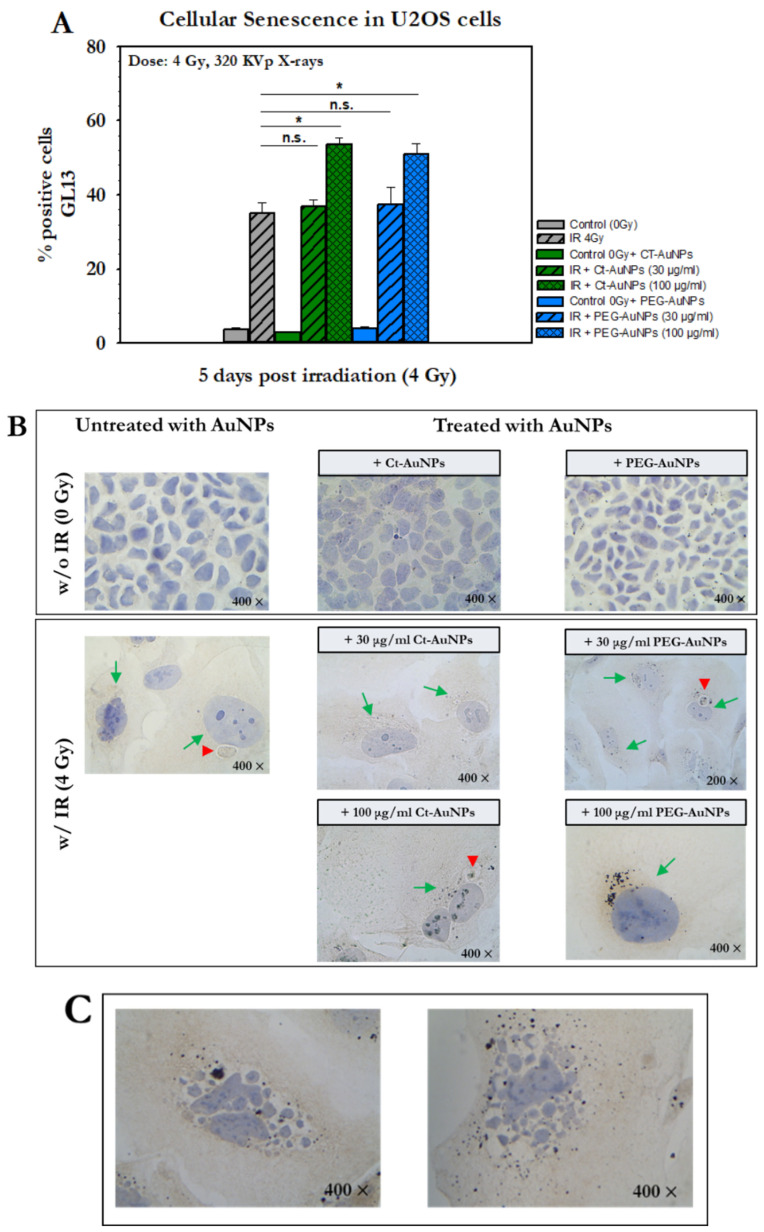
Identification of senescence in U2OS cells after treatment with AuNPs and exposure to 4 Gy, 320 KVp X-rays. (**A**) Histograms represent the mean levels of cellular senescence in each group. Statistical significance between irradiated cells with or without treatment with AuNPs was determined using Student *t* test: * *p* ≤ 0.05, n.s.: not significant. (**B**) Images represent each group with or without exposure to IR after or not the treatment with different concentrations of either 15 nm Ct-AuNPs or 5 nm PEG-AuNPs. Cells were incubated at 37 °C for 5 days after radiation exposure. Green arrows indicate senescent cells and red arrows indicate perinuclear structures of GL13 staining. Original magnifications: 200×, 400×. (**C**) Indication of increased fragmented nuclei after exposure to 4 Gy X-rays and after staining with GL13. This phenotype was more prominent in cells treated with 100 μg/mL PEG-AuNPs (5 nm) before exposure to ionizing radiation.

For this assessment and based on our previous work in detecting senescent cells with GL13 [48], we treated U2OS cells with Ct-AuNPs (15 nm) or with PEG-AuNPs (5 nm) at different concentrations (30 μg/mL and 100 μg/mL) for 24 h. Five days after radiation exposure to 4 Gy, senescent cells were detected by applying GL13 staining. Results showed that 35% of the cells irradiated but not treated with AuNPs were positive for senescence (Figure 16A).

However, the percentage of senescent cells that were treated with either 30 μg/mL or 100 μg/mL Ct-AuNPs and then exposed to 4 Gy were 37% and 53.5%, respectively, while for cells treated with 30 μg/mL or 100 μg/mL, PEG-AuNPs were 37.5% and 51%, respectively. The non-irradiated cells with and without AuNP treatment did not show any differences in the levels of senescence with the control values ranging between 2.8% and 4%. In general, no differences were observed between cells exposed only to radiation and cells that had been pre-treated with 30 μg/mL of AuNPs and then irradiated. However, the use of higher concentration of AuNPs (100 μg/mL) led to an increased number of cells stained positive for senescence (Figure 16A).

The staining pattern of GL13 was perinuclear but also extended occasionally in a larger part of the cytoplasm. The signal (detected lipofuscin) was identified as a dark-brown color of weak to moderate intensity and was also found in medium-to-large-sized perinuclear structures (red arrowheads) or in small granules diffusely distributed in the cytoplasm of senescent cells. Irradiated cells that were stained positive for senescence were usually accompanied by a senescence-associated phenotype characterized by enlarged cytoplasm and nuclei. Representative images of U2OS after GL13 staining for all groups treated with AuNPs and exposed to IR are presented in Figure 16B. Cells that were both treated with AuNPs and exposed to IR exhibited more frequently a senescent phenotype, with even more enlarged morphology and more intense granules, compared to the untreated/irradiated cells. This phenotype, however, was more abundant in cells treated with the higher concentration (100 μg/mL) of AuNPs for both 15 nm Ct-AuNPs and 5 nm PEG-AuNPs. Furthermore, a distinguishing phenotype that was observed in some of the GL13-positive senescent cells pre-treated with AuNPs and then exposed to radiation was the intense fragmentation of the nucleus (Figure 16C). Specifically, this was frequently observed in irradiated cells treated previously with 100 μg/mL PEG-AuNPs (5 nm).

## 4. Discussion

Many efforts in radiation oncology have focused on applications that aim to preferentially sensitize tumors to radiation, whilst at the same time minimizing effects in the surrounding normal tissues. One way to maximize the therapeutic ratio, meaning the differential response between a tumor and normal tissue, is through the introduction of high-atomic number (Z) material into the target. Gold (Z = 79) is a promising radiosensitizer in this regard due to its biocompatibility, high atomic number, and mass energy coefficient which, in the keV energy range, is hundreds of times greater in comparison to soft tissue, mainly due to the photoelectric effect. The result of the interaction between ionizing radiation and gold is the production of electrons, characteristic X-rays, or Auger electron emission which leads to highly localized ionizing events [7].

Apart from the radiation energy range, the type, surface coating, and AuNP concentration are all directly correlated to the biological outcome and radiation enhancement effect. In addition, it is important in such experiments to avoid or minimize toxicity, and to be able to assess carefully AuNP uptake. A previous study showed that nanoparticles with a diameter of 15 nm were nontoxic up to 75 µg/mL, although cell viability was obviously affected at concentrations of gold >150 µg/mL. At 600 µg/mL, cell viability was reduced to 41.8%, compared to 93.9% at 18.75 µg/mL [60]. Recently, Marques et al., and in the past Liu et al., also used a lower concentration of AuNPs (36 μg/mL and 0–30 μg/mL, respectively) to study their radiosensitizing effect on prostate cells and Hela cells, respectively [61,62]. Investigating the cellular localization of AuNPs can significantly help to interpret the biological outcomes after radiation. The radiosensitizing effects of AuNPs are attributed to extensive oxidative stress, DNA damage, cell cycle effects, autophagy, and ER stress [38]. Due to the short range of emitted electrons, increased DNA damage is usually attributed to AuNPs that are located inside the nucleus or near the perinuclear region [9]. On the other hand, AuNPs that are located in the cytoplasm inside vesicular structures or autophagosomes can also cause cytoplasmic stress or increased mitochondria damage due to increased oxidative stress (lipid peroxidation and protein oxidation) which might lead to apoptosis [63]. For example, previous studies showed that PEGylated AuNPs which accumulated in the cytoplasm sensitized A549 cells to X-rays through induction of apoptosis and DNA repair inhibition as a consequence of ER stress [64,65].

In this work, we treated PC3, A549, and U2OS cells with AuNP concentrations up to 100 μg/mL. Our first goal was to investigate the cellular uptake of 15 nm citrate-capped as well as 15 nm and 5 nm PEG-capped AuNPs in PC3 cells. TEM images of PC3 cells showed that a significant number of gold nanoparticles were internalized by the cells and, moreover, the uptake increased by increasing NP concentration from 10 μg/mL to 100 μg/mL. Regarding the different coating, however, the localization of AuNPs differed. Specifically, Ct-AuNPs were mostly located as agglomerates/aggregates inside membranous structures/vesicles as well as autophagosomes/autolysosomes, and even though they did not enter the nucleus, they were frequently found near the perinuclear region. This observation is in agreement with previous studies on various AuNPs’ uptake such as Ct-AuNPs [66,67,68], implying also increased autophagosome accumulation after treatment [22,69]. Even though citrate ligands are used as stabilizers, the dispersion of NPs in biological media might cause agglomeration or aggregation via physical interactions (i.e., van der Waals forces), mostly due to the presence of electrolytes or high ionic strength in the media [70,71]. Carnovale et al. study on PC3 cells also supports this opinion and their results overall are in agreement with our findings on the cellular uptake of Ct-AuNPs [72].

On the other hand, when PC3 cells were treated for 24 h with PEG-AuNPs, they had a more widespread distribution with less agglomeration, and apart from membranous structures/vesicles, the NPs were also dispersed in the cytoplasm. More importantly, both the 15 nm and 5 nm PEG-AuNPs were also able to enter the nucleus to a small extent even without having nuclear-targeting moieties. Additionally, when cells were incubated with 100 μg/mL PEG-AuNPs, the number of NPs accumulating inside the nucleus increased (see Supplementary Information, Section S4, Figure S4). Individual AuNPs have been associated also with membranous structures of the endoplasmic reticulum and the ones that were located inside the nucleus most probably had undergone endo-lysosomal escape [73]. However, comparison of the uptake quantification between 15 nm Ct-AuNPs and PEG-AuNPs showed lower overall uptake of the latter (see also Supplementary Information, Figure S5). PEGylation is a process commonly used on nanoparticles, and even though it reduces aggregates and increases the likelihood of NPs to be delivered and retained in tumor tissue, other studies report reduced uptake of PEG-coated AuNPs in various cell types including prostate cancer cells, due to retarded receptor-mediated endocytosis (RME) [74,75,76,77,78]. Wolfe et al. investigated and compared the uptake of rod-shaped Goserelin conjugated AuNPs and PEG AuNPs in PC3 cells and showed that even though both types agglomerated in the cytoplasm mostly inside vesicles, the uptake of the latter overall was 5 times lower [79]. Furthermore, PEG is one of the most frequently used surfactants on NPs since it can also achieve a more favorable treatment effect in vivo. When incorporated by the body, AuNPs will eventually be removed by the reticuloendothelial system, whereas PEGylation inhibits this reaction and may promote tumor-specific treatment [80].

Even though we showed that PEG-capped AuNPs were able to disperse in the cellular environment and even enter the nucleus, it is usually reported that NPs enter cells through the endo-lyso pathway, where, in this pathway, they are trapped either in endosomes or lysosomes and are unable to enter the cytoplasm and nucleus of cells [55]. This is also dependent on the uptake mechanisms the cell employs to internalize the NPs, such as pinocytosis or RME where, based on the latter specific types of ligands, might be placed on the surface of the NPs in order to have maximum uptake [81]. Based on our observations in this work and since NPs very often agglomerate, another mechanism we believe they use to enter the cell is through macropinocytosis, where large cellular membrane extensions or ruffles are formed as a result of cytoskeleton rearrangement, which then fuse back onto the plasma membrane, creating large vesicles (0.2–5 μm) that trap a significant amount of extracellular fluid [68,82]. Especially regarding the 15 nm Ct-AuNPs, this mechanism seemed to be the most responsible for their cellular uptake. It is also reported that citrate-capped AuNPs without functionalization, when dispersed in culture medium, might also follow the RME process through non-specific binding of the contained serum proteins on the surface of the NPs [76]. NPs can be endocytosed by both normal and cancer cells; however, some investigators report higher uptake rates of AuNPs in cancer cells compared to normal cells such as fibroblasts or hepatocytes [83,84,85], while others claim that, especially in lower concentrations, the uptake rates are similar [86].

The dose deposition is believed to be partially reliant on the number of internalized nanoparticles, meaning that the cellular uptake and distribution of AuNPs inside the cell directly influence the level of radiosensitization observed [87]. Even though DNA is the most critical target, nanoparticles were many times located next to important cellular compartments such as mitochondria or Golgi apparatus, and since the range of electrons is short, from a few nanometers to a few micrometers depending on the type of ejected electrons (photoelectrons (≤50 KeV) or Auger e^−^ (1–10 KeV)) [88,89], they might also damage these cellular targets, enhancing oxidative stress and affecting their main functions. Furthermore, in our study, we reported that AuNPs were found also inside mitotic cells. Cells in late G2 and mitosis (M-phase) are the most sensitive to radiation. This highlights the increased sensitivity that mitotic cells may have after treatment with gold nanoparticles and radiation, due to the possible closer proximity of NPs to the DNA. Consequently, targeting mitotic cells might enhance even more the effects of AuNPs after radiation.

Regarding the intracellular fate of AuNPs, we observed that NPs were localized also into or next to intracytoplasmic canaliculi (ICC), which might indicate the mechanism by which PC3 cells, after treatment with NPs, secrete NPs, away from their cellular environment. Overall, and in agreement with other studies [85,87,90], after 24 h treatment, all types of AuNPs were mostly trapped in endo-lysosomal vesicles and were still abundant inside the PC3 cells even after 48 h, although autophagosome accumulation was enhanced, especially in the case of the 15 nm Ct-AuNPs (see Supplementary Information, Figure S6).

Survival curves of PC3 and A549 cells showed increased radiosensitization after treatment with AuNPs, even though each cell line showed different levels of radiosensitization. The difference can be evaluated also in regard to the alpha and beta constants that were calculated from the data fitting. Treatment with AuNPs resulted in increased α constants and increased cell death, especially when higher concentrations of AuNPs were used. An increase in the α constant is often demonstrated after treatment with AuNPs [66,91]. PC3 cells were more radiosensitive than A549 cells with and without treatment with AuNPs since the latter had higher survival rates. Colony formation in PC3 cells was significantly reduced after treatment with AuNPs and this might be also attributed to increased complex DNA damage, as has been suggested by others [61]. Prostate tumors seem to have lower α/b ratios, where lung carcinomas exhibit higher α/β ratios leading to a more linear curve [92] and these ratios also point to the different radiosensitivity levels observed between these cell lines.

One of the major mechanisms leading to AuNP-induced radiosensitization is the induction of DNA damage. PC3, U2OS and A549 cells exhibited different levels of radiosensitization, with PC3 and U2OS being the most radiosensitive when any type of AuNPs were used, either by forming increased initial or residual γH2AX foci or both. However, their ability to form colonies was reduced after treatment with this concentration, which indicates that DNA damage is not the only stressful parameter leading to reduced cell proliferation (i.e., cell death). Interestingly, after 24 h, most AuNP-treated cells yielded more unresolved γH2AX foci compared to controls, accompanied by increased average foci intensity. Apart from the initial DNA damage [18,93,94], residual damage might be persistent when cells are treated with AuNPs and irradiated [95,96,97]. This might be due to radical interactions with water [89] or possible inhibition or delay in DNA repair [66,98] which could indicate overall increased damage complexity. Overall, the radiosensitizing effects of AuNPs were concentration-dependent; U2OS cells exhibited increased DNA damage even after 0.5 h post-IR, especially when treated with the higher concentration of nanoparticles. Citrate-capped AuNPs were avidly endocytosed, more efficiently than the PEG-modified AuNPs; however, persistent DSBs were lower in comparison to the PEG-AuNPs. This again underlines the importance of intracellular distribution, and since DNA damage is not the only reason for the induced radiosensitization, other cellular responses should also be evaluated. For example, biogenic AuNPs have been shown to sensitize A549 cells through mitochondrial membrane disruption after extensive oxidative stress [99].

Apart from the immunofluorescence assay, DNA damage was also evaluated at the chromosomal level using the G2 radiosensitivity assay [100], which has not been used so far to study AuNP-induced radiosensitization. This assay, however, gives information on a specific time window of the cell cycle, the G2 phase. We performed this experiment on PC3 cells treated with 30 μg/mL Ct-AuNPs. Even though Ct-AuNPs did not enter the cell nucleus, metaphase scoring showed a 20% increase (*p* ≤ 0.05) in the number of chromatid breaks after treatment with AuNPs, which may be attributed to the action of Ct-AuNPs near the perinuclear region. These complimentary experiments are included in the Supplementary Information, Section S5, Figure S7.

As previously mentioned, upon radiation, AuNPs can influence the cell’s functions through the generation of reactive oxygen species, DNA damage and cell cycle effects [76]. The interplay between AuNPs and the cell cycle with and without radiation has not been extensively evaluated. Nevertheless, it is known that the presence of AuNPs induces alterations in cell kinetics due to the accumulation of cells in the G2/M phase. This phase is known to be the most radiosensitive, so such accumulations lead to an overall increase in the radiosensitization [9]. The cell cycle effects after treatment with AuNPs were also evaluated both with and without exposure to IR. A549 cells did not exhibit any differences in the cell population distribution after treatment with AuNPs or after both AuNP treatment and radiation. This has been previously reported, implying that cell cycle effects might be cell-line-dependent [38]. In contrast to this opinion, Ramalingam et al. reported that treatment with 35–40 nm biogeneric AuNPs led to cell cycle arrest at the G0/G2 phase in A549 cells [99]. PC3 cells, on the other hand, showed an increased number of cells in the G2/M phase after treatment with 5 nm PEG-AuNPs, which, as indicated by other studies, may enhance radiosensitization [17]. Notably, 3 h after exposure, there was an increase in the G2/M phase and a decrease in the G1 phase compared to the IR-alone cells, for both 5 nm PEG-AuNPs and 15 nm CT-AuNPs. This has been identified also in DU-145 prostate cancer cells [15] and may also imply a prolonged G2 checkpoint at 3 h post-IR. Even after 24 h post-IR, PC3 cells still exhibited a low number of cells in the G1 phase after treatment with PEG-AuNPs. Unlike A549 cells, PC3 cells do not express p53 and DU-145 harbor mutant p53 [101], so this might explain the differences between the cell cycle profile of these two cell lines after AuNP treatment with or without radiation.

Regarding the induction of senescence, when cells were treated with the higher concentration of AuNPs (Ct-AuNPs or PEG-AuNPs) and then irradiated, the number of cells stained positive for senescence increased, which may be attributed to persistent oxidative stress or complex DNA damage. The nature of the DNA damage, in addition to its severity, can determine the cellular response (apoptosis, senescence, etc.), but also the same applies for the different cell type [102]. Based on previous studies, cells can become senescent and be detected several days (usually 3–10 days) after exposure to radiation doses 4–10 Gy [44,103,104,105]. In this work, senescence was investigated 5 days after IR exposure. Radiation-induced senescence, however, is now believed to be in some cases reversible, especially after single doses [106] and for this reason, further investigations should be made in this matter. Others also claim that induction of senescence may have advantages over apoptosis acting as a tumor suppressor mechanism [107]. Moreover, another interesting observation was the fragmented nuclei found in some GL13-positive cells, which might be an indication of mitotic catastrophe, a possible outcome of radiation-induced abnormal mitosis. As a result, and based on our observation, the biological outcomes following AuNP-induced radiosensitization should be further investigated, since different pathways of mitotic catastrophe may result in apoptosis, necrosis, senescence or even cell recovery [108].

To extend this work, we employed TEM for the first time in order to identify and quantify the DNA damage after treatment with AuNPs and exposure to IR. NPs may alter the cell’s capacity to eliminate endogenous ROS. As a consequence, NPs have been shown to induce oxidative damage to DNA, both on DNA bases and on the 2-deoxyribose moiety of the DNA backbone [109]. One of the most prominent DNA base lesions is 8-oxo-dGuo, which we detected here. After radiation, cells exhibited an increased number of DSBs, especially after treatment with the small-size 5 nm PEG-AuNPs (100 μg/mL). Additionally, double immunolocalization showed an increased number of oxidative base lesions after treatment with Ct-AuNPs. An interesting observation overall was the increased presence of clustered lesions after treatment with AuNPs and radiation. This highlights the importance in using electron microscopy to identify the possible dense ionizations near the AuNPs, especially when they are in close proximity to the DNA.

Even though cell lines are a unique tool to study the mechanisms behind the occurring radiosensitization due to AuNPs, it is crucial to discuss briefly how they might be utilized in either animal or human tumors. Depending on the type and stage of cancer, radiotherapy is usually applied in fractions giving in total 20–80 Gy [110,111]. One of the main side effects of cancer radiotherapy is that it cannot usually spare completely the nearby normal tissue. Having AuNPs inside a tumor area, we could use lower radiation doses, promote locally inside the tumor a dose enhancement effect, and at the same time, minimize detrimental effects in the surrounding normal tissue. AuNP delivery routes are another critical aspect which affects their absorption, toxicity and tissue distribution [10,112]. NPs can be administered orally, intraperitoneally, through inhalation or via intravenous and direct intratumoral injection, with the last two being the most promising [76,113]. After administration, NPs can localize in tumor tissues by using surface ligands that target specific receptors in cancer cells [114]. Moreover, unlike drugs, NPs, due to their size, are usually unable to penetrate normal vasculature and capillaries and through circulation, they preferentially accumulate and remain at tumor sites (i.e., due to porous and leaky blood vessels), as well as in inflamed tissues due to the characteristically defective architecture of the vessels [115]. This is usually referred to as the enhanced permeability and retention (EPR) effect [116].

## 5. Conclusions

Gold nanoparticles have not yet translated into the clinic, despite the large number of in vitro studies. This may be due to the disparity between predicted levels of radiosensitization based on physical action, observed biological response, and an incomplete mechanistic understanding, alongside current experimental limitations. The mechanisms behind gold nanoparticle radiosensitization are complicated and depend on many factors, such as shape, size, surface, concentration, cell type, and radiation energy. In this work, we have employed different complementary methodologies in order to fully understand the diverse biological responses of cancer cells to IR in combination with AuNPs.

Overall, our study, which focused on cancer cells, indicates that the addition of AuNPs can promote IR therapy efficacy, although this varies as a function of the cell type. Moreover, the radiosensitizing effects of AuNPs were stronger when the highest concentration of NPs (100 μg/mL) was used. Both colloidal gold solutions of Ct-AuNPs (15 nm) and PEG-AuNPs (5 nm) had good cellular uptake but the latter type of NPs also showed better distribution inside the cell and could also be located inside the nucleus as well. Additionally, as indicated by immunofluorescence, PEG-AuNPs led to an increased amount of early and late DSBs. The same results were also observed by immunogold labelling through TEM. Electron microscopy also provided us with the valuable indications that the generation of clustered DNA damage, which in general are more difficult to repair, might be a contributing factor of AuNP-induced radiosensitization. Finally, we introduced another important parameter that has not yet been investigated. Based on our findings, cellular senescence might also occur as an alternative to cell death, especially when higher concentrations of AuNPs are administered. This response has to be further investigated, in order to decipher the possible range of biological consequences and offer the optimum radiosensitization by AuNPs to cancer research. Finally, it is worth mentioning that even though PC3 and U2OS cells were found to be more radiosensitive than A549 after AuNP treatment, the cells still exhibited some differences. This highlights the different biological responses between the various types of epithelial cancer.

## Data Availability

Data are contained within the article or Supplementary Material.

## Supplementary Materials

The following supporting information can be downloaded at: https://www.mdpi.com/article/10.3390/cancers14205086/s1, Figure S1: Representative TEM electron micrographs of various types of prepared AuNPs; Figure S2: Representative flow cytometry analysis histograms of cell cycle phase distribution in A549 cells; Figure S3: Representative flow cytometry analysis histograms of cell cycle phase distribution in PC3 cells; Figure S4: Electron micrographs of PC3 cells incubated for 24 h with 5 nm PEG-AuNPs; Figure S5: Electron micrograph of a PC3 cell treated for 24 h with 30 μg/ml of PEG-AuNPs (15nm); Figure S6: Figure depicts PC3 cells, after 48 h incubation with 30 μg/ml Ct-AuNPs; Figure S7: (A) Representative metaphases for each group after G2 radiosensitivity assay. The green arrows indicate chromatid breaks and gaps that were scored in order to quantify the DNA damage. 100 metaphases were scored for each group of the irradiated cells and 50 metaphases for control groups. (B) Comparison of the average yield of chromatid breaks in PC3 cells incubated with 30 μg/mL Ct-AuNPs after irradiation with different radiation sources; Table S1: Fitted parameters of the LQ Model for experimental data shown in Figure 7a; Table S2: Fitted parameters of the LQ Model for experimental data shown in Figure 7b.
