# Supplementary material for: Biological Response of Human Cancer Cells to Ionizing Radiation in Combination with Gold Nanoparticles
Source: Cancers (Basel). 2022 Oct 17;14(20):5086. doi: 10.3390/cancers14205086 (PMC9600885; doi:10.3390/cancers14205086)
Supplement: Supplementary file 1 [file cancers-14-05086-s001.zip › cancers-1935234-supplementary.pdf]

cancers MDPI

Supplementary Information

Biological Response of Human Cancer Cells to Ionizing Radiation in Combination with Gold Nanoparticles

Supplementary Section S1. Electron Micrographs of Various Types of Prepared AuNPs

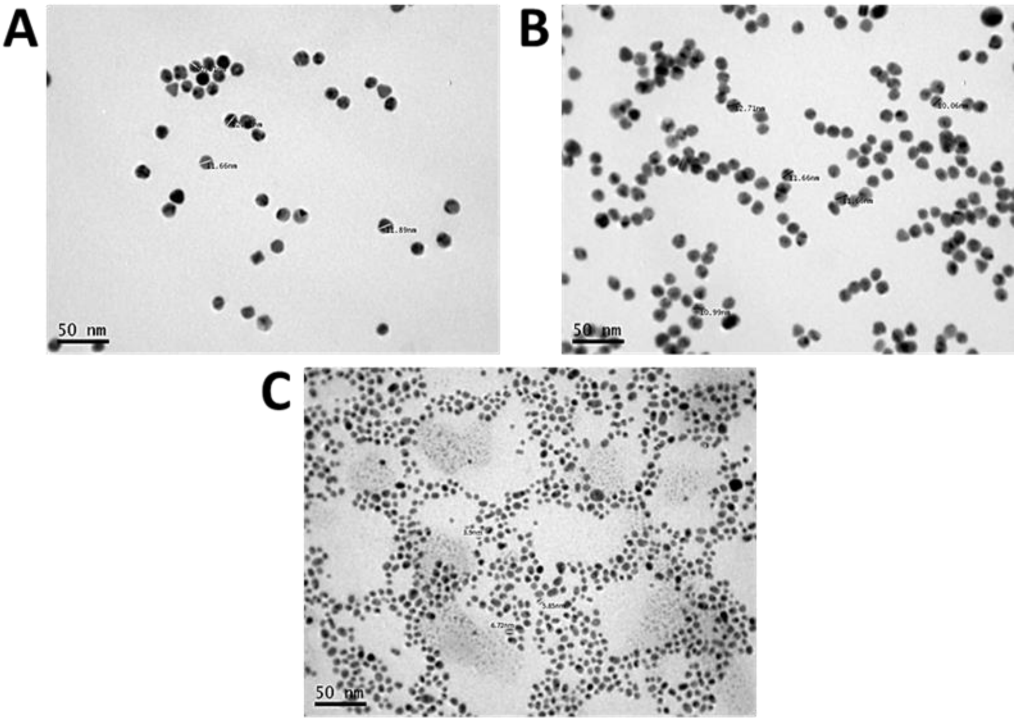

Figure S1. Representative TEM electron micrographs of various types of prepared AuNPs. **A.** Citrate-capped AuNPs, ~15 nm (Ct-AuNPs). **B.** PEG-capped AuNPs (PEG_15-AuNPs), ~15 nm. **C.** PEG-capped AuNPs (PEG-AuNPs), ~5 nm.

Supplementary Section S2. Given α and β Constants after Fitting the Data to the LQ Model

Table S1. Fitted parameters of the LQ Model for experimental data shown in Figure 7a.

**Cell line: A549**				
**Condition**	**AuNP concentration**	**α (Gy^{-1})**	**β (Gy^{-1})**	**α/β (Gy^{-1})**
IR	-	0.22 ± 0.04	0.03 ± 0.01	7.3
IR + Ct-AuNPs	30 μg/ml	$0.34 \pm .005$	0.02 ± 0.02	17
IR + PEG-AuNPs	30 μg/ml	0.41 ± 0.02	0.01 ± 0.007	41
3% AuMTA NPs	30 μg/ml	0.32 ± 0.04	0.03 ± 0.01	11

Table S2. Fitted parameters of the LQ Model for experimental data shown in Figure 7b.

**Cell line: PC3**				
**Condition**	**AuNP concentration**	**α (Gy^{-1})**	**β (Gy^{-1})**	**α/β (Gy^{-1})**
IR	-	0.22 ± 0.02	0.08 ± 0.01	2.7
IR + Ct-AuNPs	30 μg/ml	0.37 ± 0.01	0.07 ± 0.006	5.3

IR + PEG-AuNPs	30 μg/ml	0.45 ± 0.02	0.04 ± 0.009	11.2
IR + PEG-AuNPs	100 μg/ml	0.37 ± 0.05	0.09 ± 0.03	4.1
3% AuMTA NPs	100 μg/ml	0.43 ± 0.04	0.08 ± 0.02	5.4

Supplementary Section S3. Cell Cycle Distribution by Flow Cytometry for A549 and PC3 Cells

Below are representative flow cytometry histograms showing the cell cycle distribution in A549 and PC3 cells for each separate group (gated events).

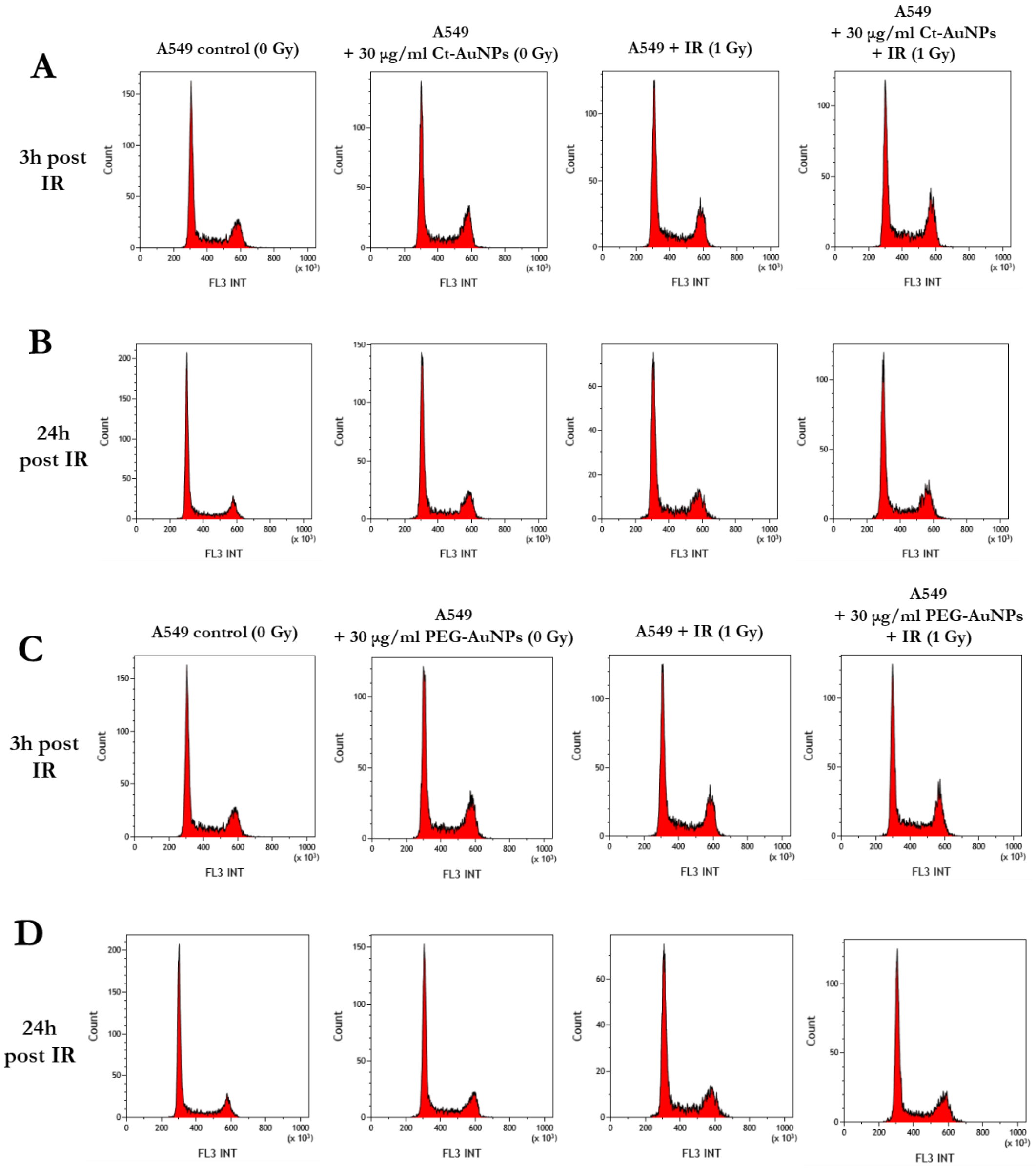

Figure S2. Representative flow cytometry analysis histograms of cell cycle phase distribution in A549 cells.

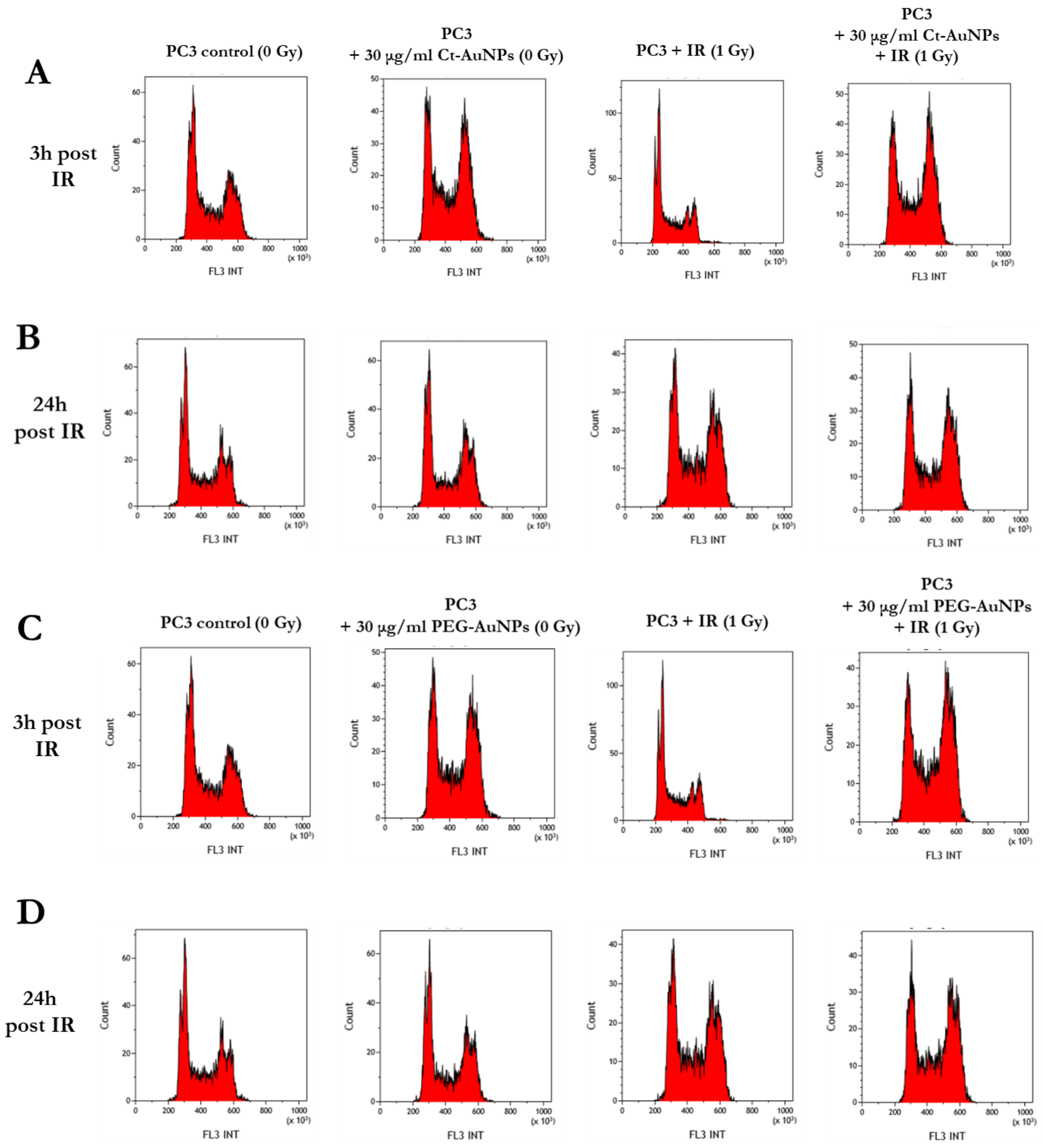

Figure S3. Representative flow cytometry analysis histograms of cell cycle phase distribution in PC3 cells.

Supplementary Section S4. Additional Representative Electron Micrographs of PC3 Cells Treated with AuNPs

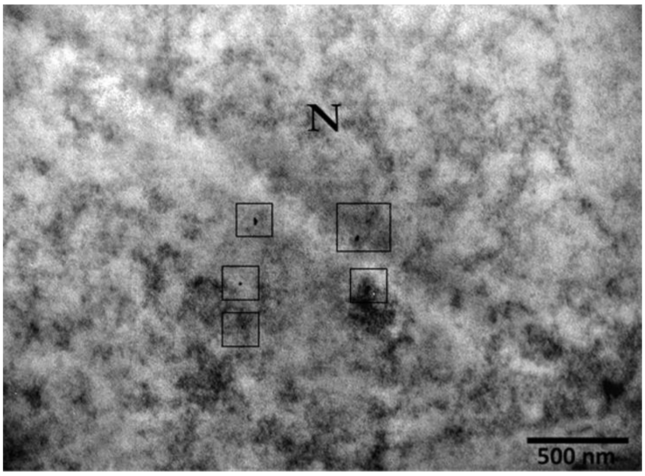

Figure S4. Electron micrographs of PC3 cells incubated for 24 h with 5 nm PEG-AuNPs. Image depicts the localization of 5 nm PEG-AuNPs inside the nucleus, after incubating cells with 100 µg/ml AuNPs. At higher concentration, small PEG-AuNPs were located inside the cell nuclei at a higher amount. Squares indicate the localized AuNPs. Scale bars: 500 nm. N: nucleus, n: nucleolus.

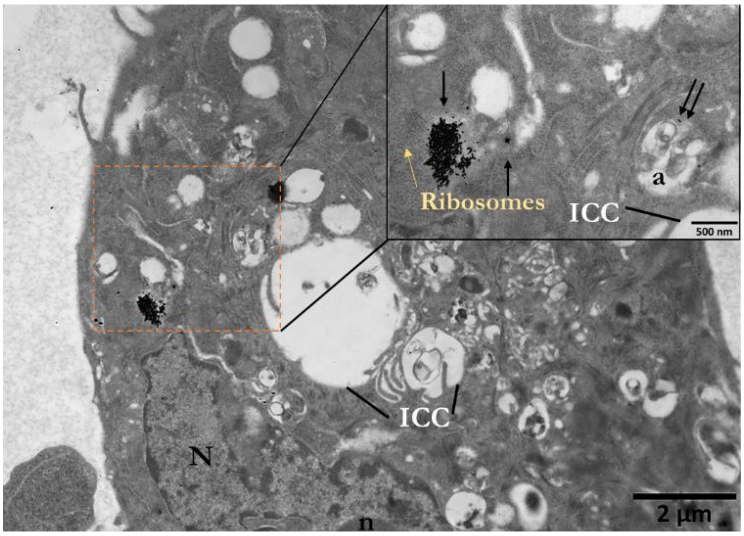

Figure S5. Electron micrograph of a PC3 cell treated for 24 h with 30 µg/ml of PEG-AuNPs (15nm). Image depicts a cell where PEG-AuNPs are concentrated in a cytoplasmic area near ribosomes (yellow arrow inset). Moreover, some NPs are localized in vesicles next to ICC indicating possible transfer into this cellular compartment. N: nucleus, n: nucleolus, a: autophagosome, ICC: intracytoplasmic canaliculus.

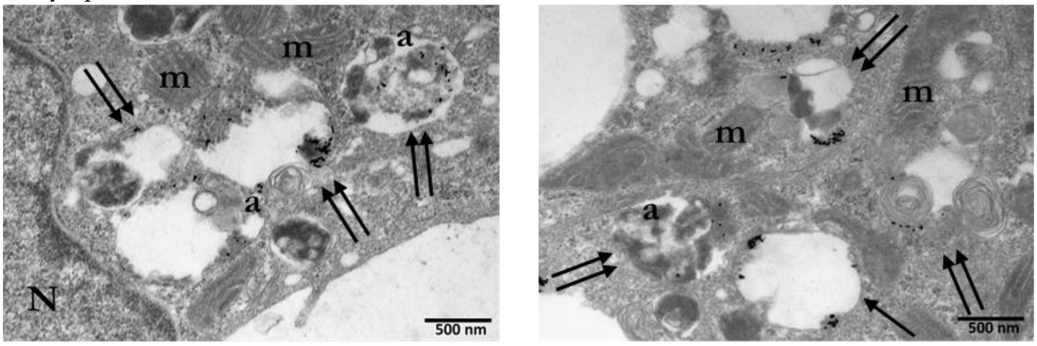

Figure S6. Figure depicts PC3 cells, after 48 h incubation with 30 μg/ml Ct-AuNPs. After 48 h autophagy is enhanced and Ct-AuNPs are located mostly inside autophagosomes and autolysosomes (double arrows). Single arrows indicate vesicular structures. N: nucleus, m: mitochondrion, a: autophagosome/autolysosome, G: Golgi apparatus, CYT: cytoplasm. Scale bars: 500 nm.

Supplementary Section S5. G2 Chromosomal Radiosensitivity Assay for the Scoring of Chromatid Breaks

Short description: Exponentially growing PC3 cells were seeded in 25-cm2 culture flasks and allowed to adhere overnight before incubation with 30 μg/ml Ct-AuNPs (15 nm) in complete culture medium for 24 h. Cells were washed thrice with PBS and incubated in complete culture medium prior to irradiation. Cells were irradiated with 1 Gy γ-rays or X-rays at room temperature, after which they were immediately placed inside the incubator at 37°C for 20 minutes and subsequently treated with 0.1 μg/ml Colcemid for 3 hours until sufficient number of mitotic cells was present. At 3 hours' time, cells were har-vested and collected by centrifugation, treated in 75mM KCl, fixed in methanol:glacial acetic acid (3:1 v/v), and processed for chromosomal aberration analysis. Standard procedures were used for chromosome preparation and staining and chromosomal damage was visualized and quantified as chromatid breaks in cells at metaphase. For each experimental point, approximately 100 cells were scored for chromatid breaks, based on standard criteria [41]. Chromatid breaks and gaps were scored, the latter only when longer than a chromatid width. Light microscopy was coupled with an image analysis system (MetaSystems, Germany) to facilitate scoring. The spontaneous aberration yield was subtracted to obtain the radiation induced G2 yield of chromatid breaks.

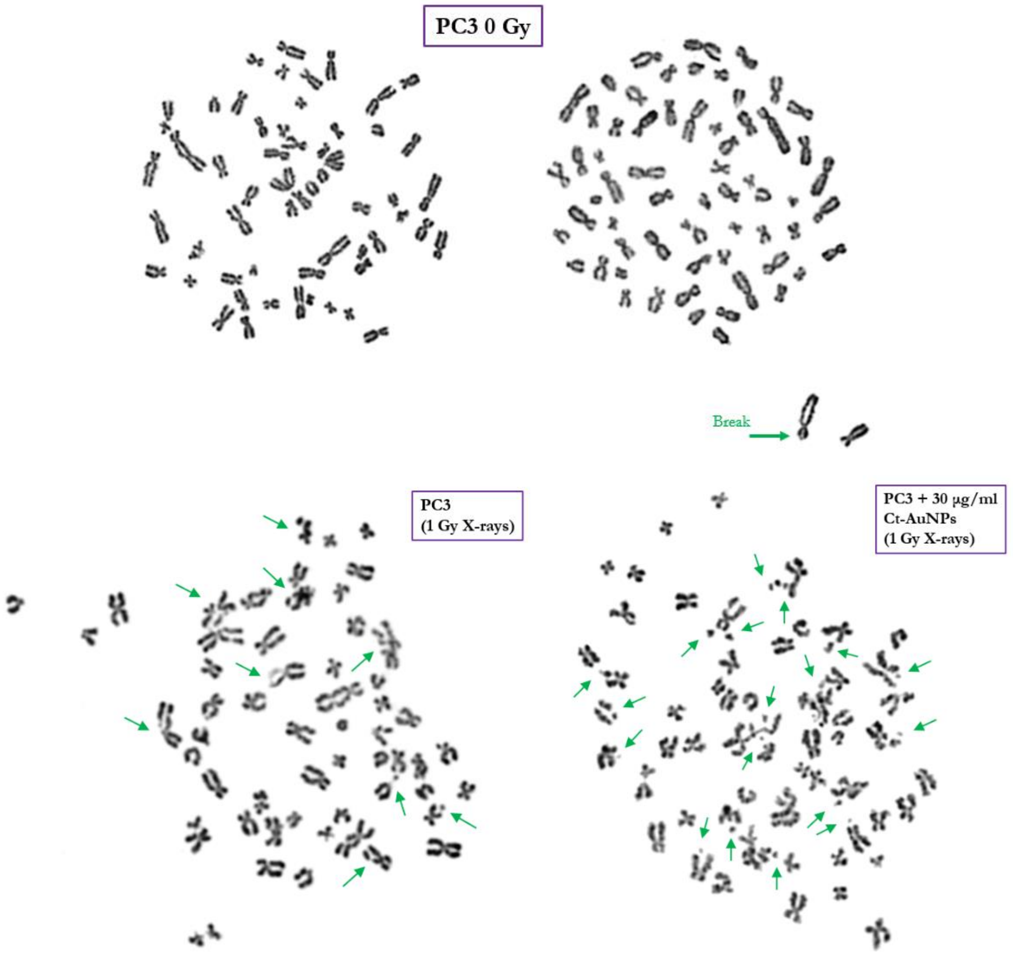

(**A**)

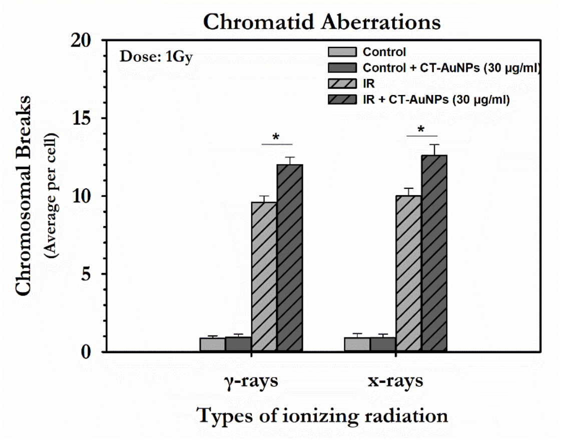

(**B**)

Figure S7. A. Representative metaphases for each group after G2 radiosensitivity assay. The green arrows indicate chromatid breaks and gaps that were scored in order to quantify the DNA damage. 100 metaphases were scored for each group of the irradiated cells and 50 metaphases for control groups. **B.** Comparison of the average yield of chromatid breaks in PC3 cells incubated with 30 μg/ml Ct-AuNPs after irradiation with different radiation sources. Histograms represent the mean ± standard deviation. Statistical significance was determined using Student t test: *P≤0.05.
